# The role of bile acids in carcinogenesis

**DOI:** 10.1007/s00018-022-04278-2

**Published:** 2022-04-16

**Authors:** Tadeja Režen, Damjana Rozman, Tünde Kovács, Patrik Kovács, Adrienn Sipos, Péter Bai, Edit Mikó

**Affiliations:** 1 Centre for Functional Genomics and Bio-Chips, Institute of Biochemistry and Molecular Genetics, Faculty of Medicine, University of Ljubljana, Ljubljana, Slovenia; 2 Department of Medical Chemistry, University of Debrecen, Egyetem tér 1., Debrecen, 4032 Hungary; 3 MTA-DE Lendület Laboratory of Cellular Metabolism, Debrecen, 4032 Hungary; 4 Research Center for Molecular Medicine, Faculty of Medicine, University of Debrecen, Debrecen, 4032 Hungary

**Keywords:** Bile acid, Primary bile acid, Secondary bile acid, Bile acid biosynthesis, Bile acid receptors, Bile acid transporters, Microbiome, CA, CDCA, DCA, LCA, UDCA, Carcinogenesis, TGR5, S1PR2, Muscarinic receptor CHRM2, Muscarinic receptor CHRM3, FXR, PXR, CAR, VDR, LXR, SHP, Oesophageal carcinoma, Gastric cancer, Hepatocellular carcinoma, Pancreatic adenocarcinoma, Colorectal carcinoma, Breast cancer, Prostate cancer, Ovarian cancer, Epithelial–mesenchymal transition, Oxidative stress, Warburg metabolism

## Abstract

Bile acids are soluble derivatives of cholesterol produced in the liver that subsequently undergo bacterial transformation yielding a diverse array of metabolites. The bulk of bile acid synthesis takes place in the liver yielding primary bile acids; however, other tissues have also the capacity to generate bile acids (e.g. ovaries). Hepatic bile acids are then transported to bile and are subsequently released into the intestines. In the large intestine, a fraction of primary bile acids is converted to secondary bile acids by gut bacteria. The majority of the intestinal bile acids undergo reuptake and return to the liver. A small fraction of secondary and primary bile acids remains in the circulation and exert receptor-mediated and pure chemical effects (e.g. acidic bile in oesophageal cancer) on cancer cells. In this review, we assess how changes to bile acid biosynthesis, bile acid flux and local bile acid concentration modulate the behavior of different cancers. Here, we present in-depth the involvement of bile acids in oesophageal, gastric, hepatocellular, pancreatic, colorectal, breast, prostate, ovarian cancer. Previous studies often used bile acids in supraphysiological concentration, sometimes in concentrations 1000 times higher than the highest reported tissue or serum concentrations likely eliciting unspecific effects, a practice that we advocate against in this review. Furthermore, we show that, although bile acids were classically considered as pro-carcinogenic agents (e.g. oesophageal cancer), the dogma that switch, as lower concentrations of bile acids that correspond to their serum or tissue reference concentration possess anticancer activity in a subset of cancers. Differences in the response of cancers to bile acids lie in the differential expression of bile acid receptors between cancers (e.g. FXR vs. TGR5). UDCA, a bile acid that is sold as a generic medication against cholestasis or biliary surge, and its conjugates were identified with almost purely anticancer features suggesting a possibility for drug repurposing. Taken together, bile acids were considered as tumor inducers or tumor promoter molecules; nevertheless, in certain cancers, like breast cancer, bile acids in their reference concentrations may act as tumor suppressors suggesting a Janus-faced nature of bile acids in carcinogenesis.

## Background

Bile acids (BAs) belong to cholesterol-derived sterols. Due to the side chain carboxyl group and hydroxylation of their steroid ring they are more polar than cholesterol. They have an amphipatic character for which they are known as natural detergents. Majority of cholesterol is excreted by bile acids that are prone to enterohepatic circulation between the gallbladder and the liver. Cholesterol absorption in the intestine and cholesterol secretion into the bile both require bile salts, which are, together with enterohepatic circulation of BAs, crucial for balancing the plasma cholesterol level [1].

BAs are also signaling molecules. They deorphanized the farnesoid X nuclear receptor (FXR) which is now known as a ligand-inducible transcription factor responsive to BAs [2]. It is important to note that BAs are metabolized in a similar manner as xenobiotics, contributing to the cross-talk between the endogenous and xenobiotic metabolism in the liver through nuclear receptors Pregnane X receptor (PXR), constitutive androstane receptor (CAR) and others [3]. While their synthesis takes place exclusively in the liver, the homeostasis and excretion involve multiple organs and compartments in the body. After discovering their signaling role, BAs have been considered as pro-carcinogenic molecules [4–6]. However, recent studies have provided evidence that in certain cancers, BAs can have antineoplastic features (e.g. breast cancer [7–11]). This novel, context-dependent, dualistic finding prompted us to thoroughly assess the involvement of BAs in carcinogenesis and cancer progression.

## Bile acid biosynthesis

**Fig. 1:**
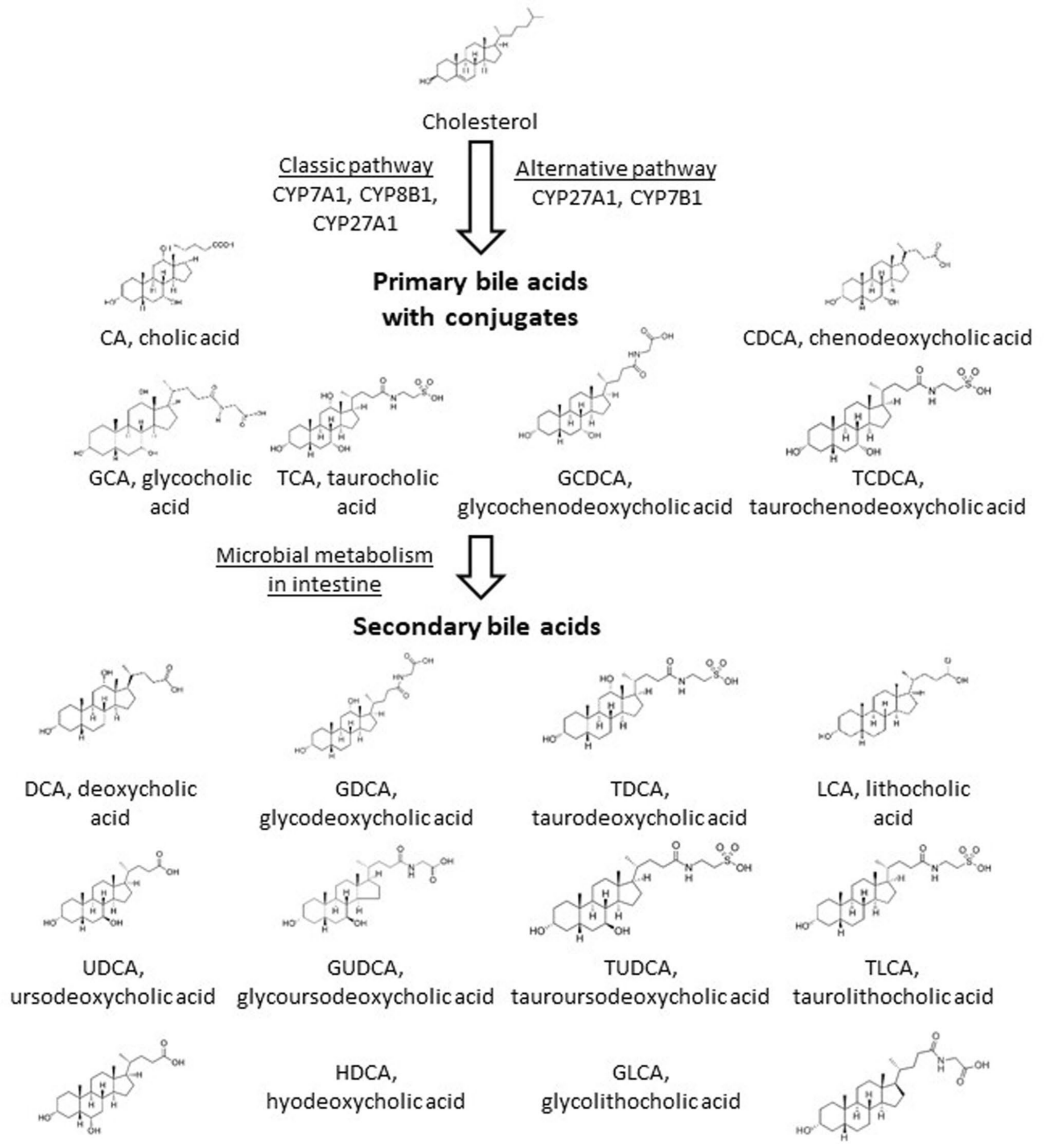
Scheme of the classical and alternative bile acids in humans. Only enzymes of the CYP family are listed while the pathway involves enzymes of other protein families. CA and DCA are conjugated and further metabolized in the intestine

The excess of free cholesterol is toxic to cells and needs to be excreted, primarily through conversion to more polar BAs. The introduction of a hydroxyl group in cholesterol reduces the half-life and directs the oxidized molecule to excretion [12]. BA synthesis is thus the main cholesterol detoxification pathway where multiple cytochrome P450 (CYP) enzymes are involved in the classical or alternative pathways (Fig. 1). The two major primary BAs in humans are cholic acid (CA) and chenodeoxycholic acid (CDCA). They are synthesized in the liver and secreted into the gallbladder as glycine or taurine conjugates [13]. The BA composition in mice substantially differs from the humans which has to be taken into account when using mouse as a model for BA related diseases. The mouse *Cyp2c70* metabolizes CDCA to more hydrophilic primary muricholic acids (MCAs) [14].

The first enzyme of the classical BA synthesis pathway is cholesterol 7α-hydroxylase (CYP7A1), leading to 7α-cholesterol in a rate-limiting reaction step, followed by several enzymatic conversions. This enzyme is prone to the negative feedback regulation by BAs and FXR [2]. Sterol 12α-hydroxylase (CYP8B1) lies at the branching point that leads to CA. Sterol 27-hydroxylase (CYP27A1) is needed for both CA and CDCA. In the alternative pathway, cholesterol is first metabolized by CYP27A1 to form 27-hydroxycholesterol that is a substrate for 25-hydroxycholesterol 7α-hydroxylase (CYP7B1) and later other enzymes [15]. The alternative pathway leads majorly to CDCA. The ratio of CA to CDCA is determined by the expression level of CYP8B1, which transforms a di-hydroxylated BA to tri-hydroxylated BA. The alternative pathway is estimated to account for about 10% of cholesterol conversion [16]. Of importance, there are major differences in individual BA synthesis genes in mouse and in humans which may be due also to different biological roles of human and mouse BA species (reviewed in [15]).

## Bacterial metabolism of bile acids, production of secondary bile acids

Hepatocytes secrete BAs to the bile canaliculi. By fusing with each other bile canaliculi form bile ducts, which eventually form the hepatic duct that runs to the gallbladder. The gallbladder empties to the duodenum upon feeding and, hence, releases BAs to the gastrointestinal tract. Primary BAs emulsify dietary fats and activate pancreatic lipases in the small bowel. BAs are then reabsorbed through the enterocytes and get to the liver for reuptake and reuse through the portal circulation. This circle is termed the enterohepatic circulation of BAs. A fraction of the reabsorbed BAs enter the systemic circulation (total BA concentration in the serum is < 5 µM in a healthy individual) and exert hormone-like effects [7, 17–20]. The reference concentrations of the serum, tissue and fecal bile acids are in Tables 1, 2, 3.

**Table 1:** Reference serum bile acid levels

	Cohort size, reference	*n* = 40 [303]		*n* = 8 [304]		*n* = 30 [305]		*n* = 28 [306]		n = 56 (pooled) serum [7]
		Mean	± SEM	Mean	± SD	Mean	± SEM	Mean	± SEM	Mean
Primary bile acids	CA	181.5	83.1	440	651	162.05	40.19	153.68	159.64	287
	GCA	233.0	56.0	85	55	42.55	13.72	72.86	93.69	301
	TCA	179.7	47.0	14	12	2.04	0.63	18.56	29.4	71
	CDCA	256.8	56.3	380	410	1160.64	299.60	654.78	660.43	563
	GCDCA	771.5	111.9	450	210	975.59	205.81	649.19	648.55	931
	TCDCA	120.2	21.8	69	56	7.51	1.74	54.28	69.18	137
Secondary bile acids	DCA	386.7	66.0	320	120	593.27	141.09	402.76	350.11	701
	GDCA	246.2	42.5	104	44	190.78	44.32	156.39	149.88	415
	TDCA	44.9	11.8	21	18	44.06	8.86	24.62	22.68	61
	LCA	12.8	1.8			9.74	1.51	94.95	57.21	31
	GLCA	16.3	4.1	17	20			25.26	15.82	25
	TLCA	23.4	3.6	0,33	0,52	0.46	0.07	22.82	19.29	
	UDCA	137.6	25.1	43	27	208.35	32.94	130.83	114.96	147
	GUDCA			76	40	60.92	9.76	128.04	178.12	330
	TUDCA	5.0	1.1	2,7	2,7	1.41	0.30	6.24	5.63	

All concentrations are in nM

*CA* Cholic acid, *CDCA* Chenodeoxycholic acid, *DCA* Deoxycholic acid, *GCA* Glycocholic acid, *GCDCA* Glycochenodeoxycholic acid, *GDCA* Glycodeoxycholic acid, *GLCA* Glycolithocholic acid, *GUDCA* Glycoursodeoxycholic acid, *LCA* lithocholic acid, *TCA* Taurocholic acid, *TCDCA* Taurochenodeoxycholic acid, *TDCA* Taurodeoxycholic acid, *TLCA* Taurolithocholic acid, *TUDCA* Tauroursodeoxycholic acid, *UDCA* ursodeoxycholic acid

**Table 2:** Reference fecal bile acid levels

	Cohort size, Reference	*n* = 97 [307]		*n* = 28 [308]		*n* = 15 [309]
		Mean µg/mg	± SD	Median nmol/g	Q1; Q3	Median ng/mg of dry feces
Primary bile acids	CA	56.16	255.46	20.19	5.03;1304.28	0.23
	GCA	199.35	317.56	2.23	1.39;3.55	
	TCA	4.14	7.82	0.72	0.46;2.11	
	CDCA	29.65	102.48	57.16	13.76;1639.92	0.23
	GCDCA			5.17	2.56;10.51	
	TCDCA	3.35	10.5	1.41	0.37;3.58	
Secondary bile acids	DCA			2159.78	1676.03;3094.08	2.6
	GDCA	110.41	167.88	2.67	1.44;6.83	
	TDCA	4.84	12.5	1.75	0.86;6.63	
	LCA	548.75	336.88	2339.24	1737.09;2782.40	3.1
	GLCA	0.18	0.18	0.91	0.41;1.28	
	TLCA	0.94	4.46	1.03	0.36;2.80	
	UDCA			17.21	8.76;33.48	0.1
	GUDCA	0.81	3.88	0.65	0.38;0.87	
	TUDCA			0.37	0.07;1.23	

*CA* Cholic acid, *CDCA* Chenodeoxycholic acid, *DCA* Deoxycholic acid, *GCA* Glycocholic acid, *GCDCA* Glycochenodeoxycholic acid, *GDCA* Glycodeoxycholic acid, *GLCA* Glycolithocholic acid, *GUDCA* Glycoursodeoxycholic acid, *LCA* lithocholic acid, *TCA* Taurocholic acid, *TCDCA* Taurochenodeoxycholic acid, *TDCA* Taurodeoxycholic acid, *TLCA* Taurolithocholic acid, *TUDCA* Tauroursodeoxycholic acid, *UDCA* ursodeoxycholic acid

**Table 3:** Reference tissue bile acid levels

		Gastric juice (µM)		Breast cyst fluid (µM)	Adipose tissue (ng/g)		Liver tissue (nmol/g)		Liver tissue (nmol/g)	
		n = 10 [310]		n = 12 [261]	n = 24 [311]		n = 6 [312]		n = 10 [313]	
		Mean	± SEM	Min–Max	Median	Min–Max	Mean	± SEM	Mean	± SEM
Primary bile acids	CA	2.38	1.09	3–119 (n = 1, ND)	˂LOD	0–11.4	21.1	13.0	30.4	5.9
	GCA	0.74	0.65		7.5	2.6–33.6				
	TCA	0.87	0.1		12.5	4.9–106.9				
	CDCA	0.03	0.04	4–305	˂LOD	˂LOD	31.0	16.0	29.8	5.4
	GCDCA	0.55	0.5		15.9	2.2–67.3				
	TCDCA	0.57	0.08		2.6	1.0–3.5				
Secondary bile acids	DCA	3.78	0.6	17–160 (n = 1, ND)	9.4	0–60.6	6.2	2.3	2.0	0.7
	GDCA	0.39	0.2		14.9	4.8–45.3				
	TDCA	5.22	0.02		4.2	1.6–6.0				
	LCA	0.12	0.02	9–23 (n = 6, ND)	˂LOD	˂LOD	1.5	0.2	0.7	0.3
	GLCA	0.12	0.007		8.1	2.9–19.0				
	TLCA	0.86	0.01		˂LOD	˂LOD				
	UDCA	0.02	0.02		˂LOD	˂LOD	2.0	0.8	1.5	0.6
	GUDCA	0.24	0.08		2.0	0–15.9				
	TUDCA	3.58	0.002		0.8	0.3–1.9				

*CA* Cholic acid, *CDCA* Chenodeoxycholic acid, *DCA* Deoxycholic acid, *GCA* Glycocholic acid, *GCDCA* Glycochenodeoxycholic acid, *GDCA* Glycodeoxycholic acid, *GLCA* Glycolithocholic acid, *GUDCA* Glycoursodeoxycholic acid, *LCA* lithocholic acid, *TCA* Taurocholic acid, *TCDCA* Taurochenodeoxycholic acid, *TDCA* Taurodeoxycholic acid, *TLCA* Taurolithocholic acid, *TUDCA* Tauroursodeoxycholic acid, *UDCA* ursodeoxycholic acid, *ND* not detected, *LOD* limit of detection

BAs are very powerful surfactants [21]; therefore, bacteria, mostly in the large bowel, need to protect themselves against being disintegrated by BAs. For example, lipopolysaccharides serve as membrane components in Gram-negative bacteria to passively ward off external toxins or BAs [22]. In addition to that, bacteria have a more sophisticated enzymatic system to cope with BAs termed BA conversion [23].

The hydroxyl groups and the tauryl or glycyl conjugate on BAs are crucial elements of the molecular structure of BAs for their strong surfactant properties. Therefore, the removal, modification or substitution of these molecular elements diminishes the potentially toxic features of primary BAs and renders them largely apolar. The dehydroxylated primary BAs are called secondary BAs and the main site for converting primary BAs to secondary BAs is the large bowel [24]. Secondary BAs can be resorbed to the portal circulation and are transported to the liver, where, however, hydroxylation and conjugation needs to be restored for reuse. The main secondary BAs in humans are lithocholic acid (LCA), deoxycholic acid (DCA) and to a lesser extent, ursodeoxycholic acid (UDCA) [24, 25].

Bile salt hydrolases (BSHs) are responsible for the deconjugation of BAs, namely the removal of glycine or taurine by breaking the C24 *N*-acyl bond. Glycine and taurine can be fed into the metabolism of bacteria to be used as an energy source [23]. BSH activity is common among the bacteria inhabiting the small and the large intestines [23]; both aerobic [26] and anaerobic bacteria can deconjugate bile salts [27]. Namely, among the Gram-positive bacteria BSH was identified in *Clostridium* [27–30]*, Enterococcus* [27, 31], *Bifidobacterium* [27, 32, 33], *Lactobacillus* [34, 35], *Streptococcus* [36], *Eubacterium* [37] and *Listeria*, among Gram-negative bacteria in *Bacteroides* [30, 38, 39], while among archea *Methanobrevibacter smithii* and *Methanosphera stadmanae* [40].

The substituents on the gonane core of BAs can be also modified, the term “secondary BA” typically stands for the removal of 7α or 7β-hydroxyl groups from primary BAs. *Clostridiales* and *Eubacteria* were shown to play a major role in dehydroxylation [23, 41–45], although other genre or species were also implicated (e.g. *Bacteroidetes*, *Escherichia)* [7, 38, 44, 46, 47]. Although BA deconjugation and dehydroxylation are different processes, they may be linked through regulatory circuits [30]. Other reactions of BAs involve oxidation, and epimerization that can be linked to intestinal *Firmicutes* (*Clostridium*, *Eubacterium*, and *Ruminococcus*), *Bacteroides* and *Escherichia* [23, 36, 37, 41, 42, 44, 45, 48]. Bacterial enzymes involved in secondary BA production are assembled in the BA inducible (bai) operon [24]. Collectively, BA transformation renders secondary BAs hydrophobic and BAs loose their ability to act as detergents or toxins to bacteria. Moreover, these changes are vital in fine-tuning the affinity of BAs to BA receptors.

Interactions between BAs and gut microbiota are bidirectional. Microbiota can transform primary BAs and, hence, modulate the composition of the BA pool [49, 50]. Inversely, BAs can influence the composition of the microbiome as well [51–56] and facilitate bacterial translocation to tissues [57], further underlining that notion BAs act as potent drivers of the early intestinal microbiota maturation [58]. Oncobiosis (dysbiosis associated with cancers) [59] can alter the secondary BA pool that may contribute to carcinogenic effects [4, 5, 7, 18]. It is of note that several other non-BA bacterial metabolites are known that play role in carcinogenesis [60–64].

## Bile acid transporters

**Fig. 2:**
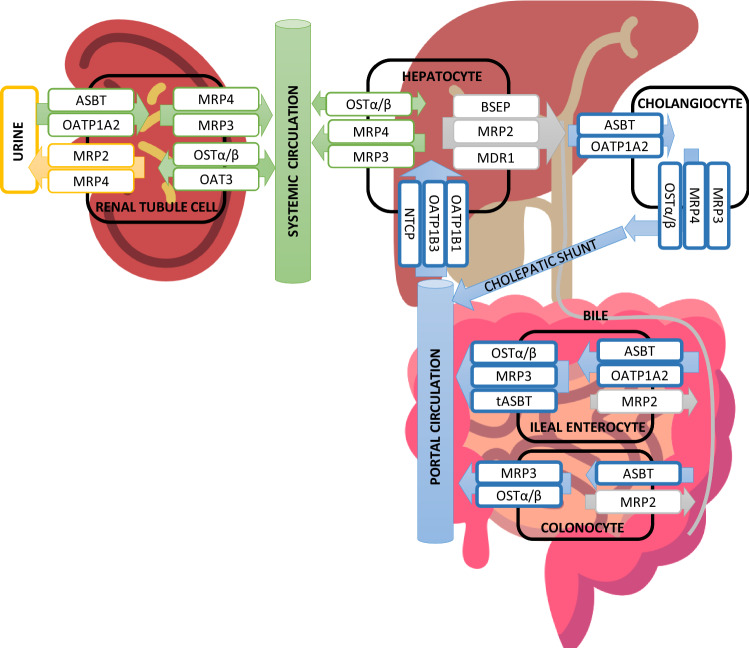
A scheme of enterohepatic and systemic circulation of bile acids and the transporters in different human cells. Transporters are coloured according to which part of the circulation they belong to. Blue are efflux and influx transporters, which transport BAs in portal circulation. Grey are efflux transporters, which contribute to bile export into bile and faeces. Green are transporters, which are responsible for BA transport into the systemic circulation. Yellow are transporters involved in the efflux of BAs into urine. *ASBT/SLC10A2* sodium-dependent bile acid transporter, *BSEP/ABCB11* ATP-dependent cassette transporter, *MRP2/ABCC2* multidrug resistance-associated protein 2, *MRP3/ABCC3* multidrug resistance-associated protein 3, *MRP4/ABCC4* multidrug resistance-associated protein 4, *OATP1A2/SLCO1A2* Solute Carrier Organic Anion Transporter Family Member 1A2, *OATP1B/SLCO1B* Solute Carrier Organic Anion Transporter Family, *SLC51A/B or OSTα/β* Solute Carrier Family members, *SLC10A2/ASBT* sodium-dependent bile acid transporter

The enterohepatic circulation of BAs depends on BA transporters in the gastrointestinal system. Almost 90% of BAs are involved in circulation due to efficient active transport [65]. Different uptake and efflux BAs transporters are present in the hepatic and intestinal cells (Fig. 2). After BAs are synthesized in the liver they are transported into the bile mainly by the ATP-dependent cassette transporter (BSEP) [65], but also minor transporters, the multidrug resistance-associated protein 2 (MRP2, ABCC2) and the multidrug resistance protein 1 (MDR1, ABCB1) [65]. From the intestinal lumen, BAs are uptaken into the intestinal cells by the major apical sodium-dependent bile acid transporter (SLC10A2, ASBT), which transports BAs also across the canalicular membrane in cholangiocytes and renal tubule apical membrane from glomerular filtrate [66]. BAs are then effluxed into the portal circulation by two Solute Carrier Family members, SLC51A or OSTα and SLC51B or OSTβ. The bile acids are then taken back up into hepatocytes by the major transporter the solute carrier family 10 (SLC10A1, NTCP), [65].

BAs can enter the systemic circulation via export across the hepatic sinusoidal membrane by OSTα/OSTβ, the multidrug resistance-associated protein 3 (MRP3, ABCC3) and the multidrug resistance-associated protein 4 (MRP4, ABCC4) [67]. The MRP transporters have a role in reducing hepatic BA concentration in cholestatic conditions. MRP3 and MRP4 are also present in cholangiocytes, where they efflux BAs to portal circulation and are part of the cholehepatic shunt together with ASBT [66]. Several transporters are expressed in the kidney, where they participate in BA elimination via urine (Fig. 2) [66, 68, 69]. The Solute Carrier Organic Anion Transporter Family, OATP1B1 or SLCO1B1 and OATP1B3 or SLCO1B3 contribute to the systemic clearance of BAs via liver [70]. Other cells also express BA transporters and can, therefore, uptake BAs from the systemic circulation [68, 69, 71].

## Bile acids as signaling molecules

**Fig. 3:**
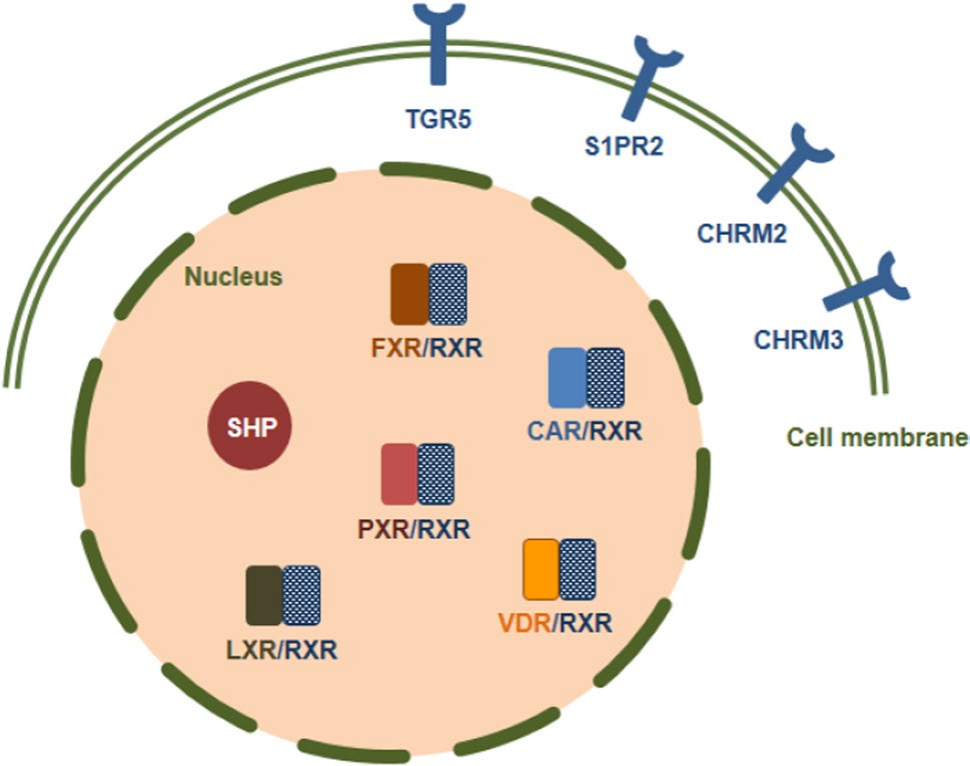
The subcellular localization of bile acid receptors. *TGR5* G protein-coupled bile acid receptor 1, *S1PR2* Sphingosine-1-phosphate receptor 2, *CHRM2* Muscarinic receptor-2, *CHRM3* Muscarinic receptor-3, *FXR* Farnesoid X receptor, *PXR* Pregnane X receptor, *CAR* Constitutive androstane receptor, *VDR* Vitamin D receptor, *SHP* Small heterodimer partner

In addition to their role in digestion, BAs act as signaling molecules. BAs can activate membrane receptors (Fig. 3), such as G protein-coupled bile acid receptor 1 (GPBAR1, also known as TGR5), sphingosine-1-phosphate receptor 2 (S1PR2), muscarinic receptors (CHRM2 and CHRM3) and nuclear receptors (NRs), such as farnesoid X receptor (FXR, NR1H4), PXR (NR1H2), vitamin D receptor (VDR, NR1H1), CAR (NR1H3) and liver X receptor (LXR, NR1H2-3). Each BA can interact with more than one receptor. Receptors are differentially activated by BAs. For example, FXR is activated by CDCA > DCA > LCA > CA [72], while TGR5 is activated by LCA > DCA > CDCA > CA [73, 74], respectively. VDR and PXR are mainly activated by LCA. BAs mediate immune responses [75], gastrointestinal mucosal barrier function, gestation [76], carcinogenesis [11, 18, 56] and metabolic diseases [20]. The activation of BA receptors may lead to the induction of signaling pathways involved in the regulation of several physiological functions, such as glucose, lipid and energy metabolism, as well as, in cancers. Below, we review the mode of action of BA receptors and highlight those receptor-mediated functions that have a key role in regulating the behavior of cancer cells.

### Cell membrane receptors

#### G protein-coupled bile acid receptor 1 (GPBAR1, TGR5)

TGR5 is a member of the G protein-coupled receptor superfamily, highly expressed in the epithelium of the gallbladder [77], the intestine [74], the brown adipose tissue and the skeletal muscle [20], as well as in the brain [78]. TGR5 is also expressed in human monocytes/macrophages [73]. TGR5 is not expressed by hepatocytes, while Kupffer cells and liver sinusoidal cells can express the receptor [79].

Secondary BAs LCA and DCA are the most potent, natural ligands for TGR5, but the receptor also responds to CDCA and CA [73, 74] and a set of artificial ligands [80–84] (Table 4). Ligand binding to the TGR5 receptor triggers activation of adenylate cyclase leading to the production of cAMP [73, 74, 85] and the downstream activation of extracellular signal-regulated kinase 1/2 (ERK1/2), protein kinase A (PKA), protein kinase B (AKT), mammalian target of rapamycin complex 1 (mTORC1) and Rho kinase [86–89]. TGR5 activation leads to metabolic changes characterized by energy expenditure and β-oxidation [20, 90]. BA-dependent induction of TGR5 has immunomodulating effects. Most studies point to TGR5-dependent immunosuppression [73, 79, 91–94] partly due to the suppression of the Toll-Like Receptor 4—Nuclear factor-κB (TLR4–NF‐κB) pathway [91, 93, 94]. In line with that, in a murine model of breast cancer, LCA treatment induced the proportions of tumor-infiltrating lymphocytes through TGR5 [7].

**Table 4:** Bile acid receptors, their ligands and connected cancers

Receptor	Bile acid ligands	Connected cancers
GPBAR1 (TGR5)	TLCA, LCA, DCA, CDCA, CA	Breast cancer Pancreatic cancer Gastric cancer Colon cancer Oesophageal adenocarcinoma
S1PR2	GCA, TCA, GCDCA, TCDCA, GDCA, TDCA	Cholangiocarcinoma Oesophageal adenocarcinoma
CHRM2, CHRM3	LCT, TLCA	Colon cancer Cholangiocarcinoma
FXR	CDCA, DCA, LCA, CA	Colon cancer Hepatocellular carcinoma Breast cancer Oesophageal adenocarcinoma
PXR	LCA, 3-keto-LCA, CDCA, DCA, CA	Colon cancer Oesophageal adenocarcinoma
CAR	LCA	Breast cancer
VDR	LCA	Colon cancer
LXR α/β	HDCA	Ovarian cancer
SHP	DCA	Hepatocellular carcinoma Breast cancer Gastric cancer

*CA* Cholic acid, *CAR* Constititive androstane receptor, *CDCA* Chenodeoxycholic acid; CHRM2/M3, Muscarinic receptor 2 and 3, *DCA* Deoxycholic acid, *FXR* Farnesoid X receptor, *GCA* Glycocholic acid, *GCDCA* Glycochenodeoxycholic acid, *GDCA* Glycodeoxycholic acid, *HDCA* hyodeoxycholic acid, *LCA* Lithocholic acid, *LCT* Lithocholyltaurine, *LXR* Liver X receptor, *PXR* Pregnane X receptor, *S1PR2* Sphingosine-1-phosphate receptor 2, *SHP* Small heterodimer partner, *TCA* Taurocholic acid, *TCDCA* Taurochenodeoxycholic acid, *TDCA* Taurodeoxycholic acid, *TGR5/GPBAR1* G protein- coupled bile acid receptor 1, *TLCA* Taurolithocholic acid, *VDR* Vitamin D receptor

#### Sphingosine-1-phosphate receptor 2 (S1PR2)

Conjugated BAs activate S1PR2 [95–97] that upregulates the expression of sphingosine kinase 2 (SphK2), which in turn enhances the level of sphingosine-1-phosphate in the nucleus. Elevated nuclear sphingosine-1-phosphate inhibits the function of histone deacetylases resulting in the upregulation of genes encoding nuclear receptors and enzymes involved in lipid and glucose metabolism [98] Similar to TGR5, ligand binding to S1PR2 can activate different downstream signaling pathways, such as ERK, AKT and/or c-Jun N-terminal kinase (JNK1/2) [96, 97, 99, 100]. Glycochenodeoxycholic acid (GCDCA) can trigger apoptosis in hepatocytes through activating S1PR2 [101]. S1PR2 is highly expressed in macrophages [102] and has widespread immunological roles [100, 102, 103].

#### Muscarinic receptors (CHRM2 and CHRM3)

Taurine conjugated BAs can activate muscarinic receptors, the cholinergic receptor muscarinic 2 and 3 (CHRM2 and CHRM3). CHRMs are overexpressed in colon cancer cells and stimulate cell proliferation and invasion [104, 105]. Taurolithocholic acid (TLCA) induces cholangiocarcinoma cell growth via muscarinic acetylcholine receptor and EGFR (epithelial growth factor receptor)/ERK1/2 signaling [106].

### Nuclear receptors

#### Farnesoid X receptor (FXR, NR1H4)

FXR is a member of the nuclear hormone receptor superfamily. There are two FXR genes, encoding FXRα and FXRβ of which only FXRα is expressed, FXRβ is present as a non-expressed pseudogene in humans. The FXR receptor heterodimerizes with retinoid X receptor (RXR) and binds to FXR response elements (FXREs) within the regulatory regions of its target genes [107]. BAs are physiological ligands for FXR (with decreasing affinity: CDCA, DCA, LCA, CA) [72]. FXR is expressed mainly in the liver, intestine, kidney and adrenal glands [107].

FXRα controls BA synthesis, transport and detoxification. The activation of FXR receptor by BAs reduces the expression of *Cyp7a1* and *Cyp8b1*, key enzymes of BA biosynthesis pathway. In the liver, FXRα induces the transcription of its target gene encoding small heterodimer partner (SHP, NR5O2), an orphan nuclear hormone receptor (see in detail later) that lacks a DNA binding domain and acts as a transcriptional repressor [108]. SHP inhibits the expression of *Cyp7a1* through the inhibition of the interaction with liver receptor homolog-1 (LRH-1, NR5A2) [109]. In addition to LRH-1, SHP also prevents the function of hepatocyte nuclear factor-4α (HNF4α), a positive regulator of *Cyp7a1* and *Cyp8b1* [110]. In the intestine, FXRα induces the expression of fibroblast growth factor 19 (FGF19) in humans and its mouse homolog fibroblast growth factor 15 (FGF15). The secreted growth factor via portal blood reaches the liver where it binds to its receptor, fibroblast growth factor receptor 4 (FGFR4) and induces JNK and ERK pathways and causes repression of *Cyp7a1*, thus reducing BA synthesis [111]. In addition to *Cyp7a1*, *Cyp8b1* is also repressed by FXRα via SHP-dependent mechanism involving HNF4α [110].

FXRα is also a key regulator of BA transport by influencing the expression of BA transporters. FXRα activation suppresses BA reuptake to hepatocytes through repressing the expression of *NTCP* via SHP dependent mechanism [112]. At the same time, FXRα facilitates the efflux of BAs from hepatocytes into bile by enhancing the expression of *BSEP* and into the systemic circulation via *OSTα/β* transporter [113]. FXR also upregulates MRP2, which promotes BA secretion into the gallbladder. Finally, FXRα activates the expression of intestinal BA-binding protein (I-BABP) in the ileum which promotes transport of BAs from enterocytes into portal blood [114] whereas limits enterocyte uptake of BAs by reducing *ASBT* expression. FXRα increases the expression of enzymes involved in the detoxification of BAs, such as cholesterol 25-hydroxylase or cytochrome P450 family 3 subfamily A4 (CYP3A4) [115], dehydroepiandrosterone-sulfotransferase (SULT) 2a1 [116] and uridine 5′-diphosphate-glucuronosyltransferase 2B4 (UGT2B4) [117]. Many studies have reported the relationship between FXR and inflammation. NF-kB activation suppressed FXR-mediated gene expression, indicating that there is a negative crosstalk between the FXR and NF-kB signaling [118].

#### Pregnane X receptor (PXR, NR1I2)

In humans, PXR is mainly expressed in the liver and intestine [119]. Among BAs, the most potent ligand of PXR is LCA, and the oxidized, 3-keto form of LCA. PXR acts as a xenobiotic sensor and regulates the expression of genes involved in the detoxification and metabolism of BAs [120]. Upon ligand binding, PXR binds to the promoter of its target gene as a heterodimer with RXR. Activation of PXR induces the uptake of xenobiotics, their modification by phase I enzymes (CYPs, including CYP3A, CYP2B, CYP2C), conjugation by phase II enzymes, such as glutathione S-transferases, UDP-glucuronosyl-transferases (UGTs) and sulfotransferases, and finally elimination by phase III drug transporters including MDR1, MRP2 and organic anion-transporting polypeptide (OATP2) [120]. The activation of PXR prevents cholesterol gallstone disease by regulating BA biosynthesis and transport [121] and protects the liver against LCA-induced toxicity [122–125]. PXR activation disrupts the interaction between HNF4α and peroxisome proliferator-activated receptor gamma coactivator 1 alpha (PGC-1α, PPARGC1A), which is required for the activation of CYP7A1 gene expression, thus reducing the expression of *CYP7A1* and inhibiting the synthesis of BAs [126]. PXR activation is anti-inflammatory [127–129]. PXR activation facilitates lipogenesis, suppressing β-oxidation and ketogenesis and gluconeogenesis [130–132]. Furthermore, PXR through HNF4 and PGC-1α modulates the expression of *CYP7A1* [133].

#### Constitutive androstane receptor (CAR, NR1I3)

CAR is the closest relative to the PXR and is expressed primarily in the liver. First studies identified that CAR has constitutive transcriptional activity in the absence of its ligand [134]. Later, it was reported that the constitutive transcriptional activity of CAR is reversed by androstane metabolites, which are inverse agonists [135]. CAR can be activated by direct ligand binding and indirect activation [136]. In the absence of ligand binding, CAR forms a heterodimer with RXR and transactivates its target genes [137]. CAR recruits coactivators in the nucleus, such as steroid receptor coactivator 1 (SRC-1, NC0A1) and PGC-1 [138]. Similar to PXR, CAR controls the expression of drug-metabolizing enzymes and transporters, thereby supporting the detoxification of xenobiotics [120, 139]. In contrast to PXR, it remains unclear whether BAs can function as natural ligands for CAR; nevertheless, there are reports underscoring the involvement of CAR in BA signaling [11].

#### Vitamin D receptor (VDR, NR1I1)

In humans, VDR is highly expressed in the kidney, intestine, bone as well as in hepatocytes but expressed at low levels in other tissues [140–142]. LCA is a potent endogenous VDR ligand [143, 144]; hence, VDR can act as an intestinal BA sensor. VDR activation induces expression of *CYP3A* that metabolizes LCA [143, 145]. In addition, VDR induces the expression of *SULT2A1*, *MRP3* and *ASBT* to stimulate BA sulfonation, excretion and transport [146–148]. The activated VDR plays a role in the inhibition of BA synthesis via suppression of *CYP7A1*, thus protecting liver cells during cholestasis [140].

VDR can function as a nuclear receptor and a membrane-bounded receptor. Upon ligand binding, VDR translocates into the nucleus, where it binds to DNA response elements as a heterodimer with RXR to mediate gene transcription. Plasma membrane-associated VDR receptor activates several signaling cascades to inhibit *CYP7A1* transcription [142, 149]. It has been shown that the activation of membrane VDR signaling by LCA in the liver activates MEK1/2ERK1/2 pathway, which stimulates nuclear VDR/RXRα heterodimer recruitment of corepressors to inhibit *CYP7A1* gene transcription [150]. In biliary epithelial cells, bile salts (CDCA, UDCA) stimulate the expression of cathelicidin, an antimicrobial peptide, via VDR and FXR to control innate immunity [151]. The possible role of VDR in regulating immunity and the role of VDR in different cancer cells and diseases is reviewed in detail elsewhere [152].

#### Liver X receptor (LXR, NR1H2-3)

LXRs are activated by naturally occurring cholesterol metabolites such as oxysterols and bind to DNA as heterodimers with the RXR [153]. LXRα (NR1H3) and LXRβ (NR1H2) share a high structural homology [154]. LXRβ is ubiquitously expressed, while LXRα is primarily expressed in the liver, the adipose tissue, the intestine and macrophages. Upon ligand activation LXRs regulate gene expression via binding to LXR response elements in the promoter regions of the target genes. LXRα promotes the conversion of cholesterol into BAs through the induction of CYP7A1 expression in the liver. LXRs enhance the efflux of cholesterol from cells [155] and have an anti-inflammatory response in the adipose tissue and macrophages [156]. Hyodeoxycholic acid (HDCA), a naturally occurring secondary BA generated by bacterial C-6 hydroxylation of LCA, is a weak LXRα agonist [157].

#### Small heterodimer partner (SHP, NR5O2)

SHP is a unique nuclear receptor that contains a ligand-binding domain but lacks the conserved DNA-binding domain. SHP acts as a transcriptional corepressor regulating different metabolic processes, including lipid, glucose, energy homeostasis and BA synthesis via interaction with multiple transcription factors and nuclear receptors (reviewed in [158]). BAs or FGF19 signaling enhances posttranslational modifications of SHP, which modulates the regulatory function of SHP protein [159, 160]. SHP acts as an inhibitory regulator in Hedgehog/Gli signaling pathway [161].

## Effects of bile acids in cancers

**Fig. 4:**
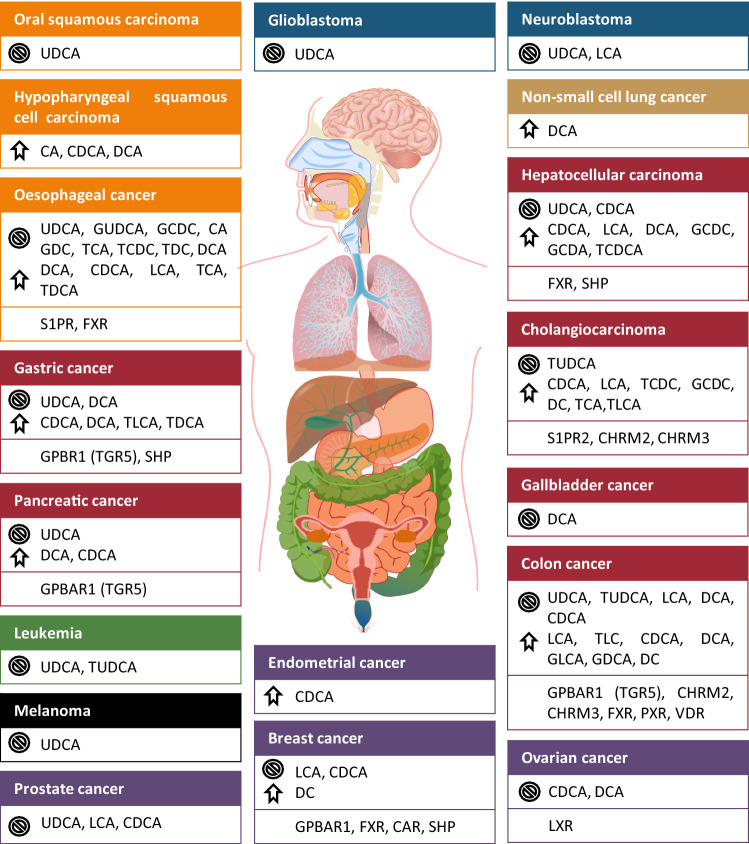
Different roles of bile acids and bile acids receptors in a wide variety of cancers. Some BAs have opposite effects, which depend on the cell line, BA concentration and other treatment conditions. The crossed circle symbol marks the tumor suppressor effects and the arrow marks the tumor promoter effects. *CA* Cholic acid, *CAR* Constititive androstane receptor, *CDCA* Chenodeoxycholic acid, *CHRM2/M3* Muscarinic receptor 2 and 3, *DC* Deoxycholate, *DCA* Deoxycholic acid, *FXR* Farnesoid X receptor, *GCDA* Glycochenodeoxycholate acid, *GCDC* Glycochenodeoxycholate, *GDC* Glycodeoxycholate, *GDCA* Glycodeoxycholic acid, *GLCA* Glycolithocholic acid, *GUDCA* Glycoursodeoxycholic acid, *LCA* Lithocholic acid, *PXR* Pregnane X receptor, *S1PR2* Sphingosine-1-phosphate receptor 2, *SHP* Small heterodimer partner, *TCA* Taurocholic acid, *TCDC* Taurochenodeoxycholate, *TCDCA* Taurochenodeoxycholic acid, *TDC* Taurodeoxycholate, *TDCA* Taurodeoxycholic acid, *TGR5/GPBAR1* G protein- coupled bile acid receptor 1, *TLC* Taurolithocholate, *TLCA* Taurolithocholic acid, *TUDCA* Tauroursodeoxycholic acid, *UDCA* Ursodeoxycholic acid, *VDR* Vitamin D receptor

The role of BAs was implicated in a wide variety of neoplasias (Fig. 4, Tables 5, 6, 7). When assessing the effects of BAs, one has to keep in mind that the concentrations applied in the experiments need to correspond to the reference concentrations in serum or the compartment in question (e.g. parts of the gastrointestinal tract). However, several reports are using substantially higher concentrations than the reference. These studies need to be considered as ones using “therapeutic” concentrations. In the forthcoming chapters, we will review those neoplasias where BAs were implicated in pathogenesis.

**Table 5:** Tumor suppressive effects of UDCA, TUDCA and GUDCA in cancers

Cancer type	Cell models	Concentration	Effects	Ref
Glioblastoma	A172, LN229	400–800 µM	UDCA inhibits cell viability, induces ROS production and endoplasmic reticulum stress, synergizes with proteasome inhibitor Bortezomib	[314]
Neuroblastoma	SH-SY5Y	100 µM	TUDCA protects against mitochondrial damage, cell death and ROS generation via mitophagy	[315]
Pancreatic cancer	HPAC, Capan1	0.2 mM	UDCA reduces intracellular ROS level and *Prx2* expression, as well as suppresses EMT and stem cell formation	[227]
Prostate cancer	DU145	0–200 µg/ml	UDCA inhibits cell growth and induces apoptosis via extrinsic and intrinsic pathways	[274]
Melanoma	M14, A375	0–300 µg/ml	UDCA inhibits cell proliferation and induces apoptosis via ROS-triggered mitochondrial-associated pathway	[316]
Hepatocellular carcinoma (HCC)	Huh-BAT, HepG2	750 µM	UDCA has a synergistic effect on the antitumor activity of sorafenib in HCC cells via activation of ERK and dephosphorylation of STAT3	[195]
	HepG2, BEL7402	0.1–1 mM	UDCA inhibits proliferation and induces apoptosis of HCC cell lines by blocking cell cycle and regulating the expression of *Bax/Bcl-2* genes. UDCA suppresses growth of BEL7402 cells in vivo	[196] [317]
	HepG2	0.25–1 mM	UDCA induces apoptosis via regulating of *Bax to Bcl-2* ratio, the expressions of *Smac* and *Livin*, and caspase-3 expression and activity	[197]
	Huh-Bat, SNU761, SNU475	200 µM	UDCA suppresses cell growth and induces DLC1 tumor suppressor protein expression by inhibiting proteasomal DLC1 degradation in an ubiquitin-independent manner	[198]
	HepG2, SK-Hep1, SNU-423, Hep3B	100 µM	UDCA switches oxaliplatin-induced necrosis to apoptosis via inhibition of ROS production and activation of the p53-caspase 8 pathway	[199]
Oral Squamous Carcinoma	HSC-3	100–400 µg/ml	UDCA induces apoptosis via caspase activation	[318]
Leukemia	T leukemia cell line (Jurkat cell)	100 µg/ml	TUDCA and UDCA induce a delay in cell cycle progression	[319]
Gastric cancer	MKN‑74	200 µM	UDCA suppresses chenodeoxycholic acid-induced PGE2 production and tumor invasiveness without affecting the *COX-2* expression	[320]
	SNU601, SNU638	0.25–1 mM	UDCA induces apoptosis, which is mediated by lipid raft-dependent death receptor 5 (DR5) expression and activation	[321]
	SNU601	0.6–1 mM	UDCA induces apoptosis via MEK(MAPK)/ERK pathway. DCA-mediated ERK activation exerts an antiapoptotic activity in this cell line	[322]
	SNU601	0.5–1 mM	UDCA induces apoptosis via CD95/Fas death receptor, downregulates ATG5 level and prevents autophagic pathway	[323]
Oesophageal cancer / Barett’s esophagus	BAR-T, BAR-10 T	125–250 µM	UDCA increases antioxidant expression and prevents DCA-induced DNA damage and NF-κB activation	[324]
	SKGT-4, OE33	300 µM	UDCA inhibits DCA-induced NF-κB, AP-1 activation and *COX-2* upregulation	[325]
	BE CP-A	0.1–0.2 mM	GUDCA has cytoprotective role by inhibiting oxidative stress	[326]
Colon cancer	HCT116	500 µM	UDCA inhibits DCA-induced apoptosis via modulation of EGFR/Raf-1/ERK signaling	[246]
	HCT116	500 µM	UDCA suppresses DCA-induced apoptosis by stimulating AKT-dependent survival signaling	[327]
	HCT116	500 µM	UDCA protects colon cancer cells from apoptosis induced by DCA by inhibiting apoptosome formation independently of the survival signals mediated by the PI3K, MAPK, or cAMP pathways	[328]
	HCT116	400 µM	UDCA inhibits cell proliferation by suppressing the expression of c-Myc protein and cell cycle regulatory molecules	[329]
	HT29, HCT116	0.2 mM	UDCA inhibits cell proliferation by regulating ROS production, induces activation of ERK1/2, and inhibits formation of colon cancer stem-like cell	[244]
	HCT116	300 µM	UDCA inhibits interleukin β1 and blocks DCA-induced NF-κB and AP-1 activation	[330]
	HT-29	250 µM	UDCA suppresses cell growth, which is enhanced in the presence of caveolin; UDCA promotes endocytosis and degradation of EGFR receptor	[331]
	HCT116, COLO 205	50 µg/ml	TUDCA suppresses NF-κB signaling and ameliorates colitis-associated tumorigenesis	[332]
Cholangiocarcinoma	Mz-ChA-1	0.2–200 µM	TUDCA inhibits cell growth via a signal-transduction pathway involving MAPK p42/44 and PKCα	[333]

*AKT* AKT Serine/Threonine Kinase 1, *AP-1* activator protein-1, *ATG5* Autophagy Related 5, *BIRC7/Livin* baculoviral IAP repeat-containing protein 7, *Bax* Bcl-2-associated X protein, *Bcl-2* B-cell lymphoma 2, *cAMP* Cyclic adenosine monophosphate, *c-Myc* Myc-Related translation/localization regulatory factor, *COX2* cyclooxygenase-2, *DCA* Deoxycholic acid, *Dlc1* Deleted in Liver Cancer 1, *DR5* death receptor 5, *EGFR* epithelial growth factor receptor, *EMT* epithelial–mesenchymal transition, *ERK* extracellular signal-regulated kinase, *FAS/CD95* Fas Cell Surface Death Receptor, *GUDCA* Glycoursodeoxycholic acid, *HCC* hepatocellular carcinoma, *MAPK* mitogen-activated protein kinase, *NF-κB* nuclear factor κappa-light-chain-enhancer of activated B cells, *PGE2* prostaglandin E2, *PI3K* Phosphatidylinositol 3-kinase, *PKCα* protein kinase C α, *Prx2* peroxiredoxin II, *RAF1* Raf-1 Proto-Oncogene, Serine/Threonine Kinase, *ROS* reactive oxygen species, *Smac* second mitochondria-derived activator of caspase, *STAT3* signal transducer and activator of transcription 3, *TUDCA* Tauroursodeoxycholic acid, *UDCA* Ursodeoxycholic acid

**Table 6:** Antitumor effects of bile acids other than UDCA in cancers

Cancer types	Cell lines	Concentration of bile acids	Effects of bile acids	Refs.
Breast cancer	MCF7, MDA-MB-231	LCA (50–200 µM)	LCA induces *TGR5* expression and exhibits anti-proliferative and pro-apoptotic effects. LCA inhibits lipogenesis and reduces *ERα* expression in MCF7 cells	[10]
	MCF7, 4T1	LCA (0.3 µM)	LCA inhibits cell proliferation, EMT transition, VEGF production and induces antitumor immune response and elicits changes in metabolism through TGR5 receptor	[7]
	MCF7, 4T1	LCA (0.3 µM)	LCA induces NRF2/NFE2L2 dependent oxidative/nitrosative stress via TGR5/CAR receptors	[11]
	MCF7	CDCA (50 µM)	CDCA activates FXR receptor and inhibites Tamoxifen-resistant breast cancer cells proliferation and EGF-induced growth through downregulation of *HER2* expression	[268]
	MCF7, MDA-MB-231	CDCA (30 µM)	CDCA induces cell death via activation of FXR	[334]
Colon cancer / Colorectal carcinoma	Caco-2, HT29C19A	LCA (20 µM)	LCA activates VDR to block inflammatory signals in colon cells	[335]
	HCT116	LCA (150–400 µM)	LCA activates p53 and promotes apoptosis by its bindig to MDM4 and MDM2, key negative regulators of p53	[336]
	HCT116	DCA, CDCA (500 µM)	DCA and CDCA induce apoptosis	[337]
	HCT116	DCA (200–250 µM)	DCA induces apoptosis via AP-1 and C/EBP mediated GADD153 expression	[338]
	HCT116	DCA (0.05–0.3 mM)	DCA in physiologically relevant dose inhibits cell growth and induces apoptosis	[242]
Gallbladder cancer (GBC)	NOZ, GBC-SD, EGH1	DCA (50–200 µM)	DCA functions as a tumor suppressive factor in GBC by interfering with miR-92b-3p maturation	[339]
Gastric cancer	SGC7901	DCA (0.1–0.3 mM)	DCA induces apoptosis via the mitochondrial-dependent pathway	[186]
	BGC-823	DCA (0.3 mM)	DCA inhibits the growth of gastric cancer cells via p53 mediated pathway	[185]
	SNU-216, MKN45	DCA (200 µM)	DCA induces *MUC2* expression and inhibits tumor invasion and migration	[340]
Hepatocellular carcinoma (HCC)	HEPG2, L02	CDCA (10–50 µM)	CDCA reduces the expression of inflammation mediators, inhibits STAT3 phophorylation and increases expression of *SOCS3* via FXR	[193]
	HepG2, Huh7, mouse hepatoma Hepa 1–6	CDCA (50–100 µM)	CDCA induces tumor suppressor N-Myc downstream regulated gene 2 (NDRG2) expression through FXR receptor	[194]
Neuroblastoma (NB)	SK-n-MCIXC, BE(2)-m17, SK-n-SH, Lan-1	LCA (100 µM)	LCA selectively kills the NB cell lines while sparing normal neuronal cells. LCA triggers intrinsic and extrinsic pathways of apoptosis	[8]
Ovarian cancer	OVCAR3	CDCA, DCA (10 µM)	CDCA and DCA upregulate *BRCA1* and downregulate *ER1* gene expression, which are important implications for disease penetrance and chemoprevention strategies in carriers of *BRCA1* mutations	[281]
	A2780	CDCA, DCA (200–400 mM)	CDCA and DCA have significant cytotoxic activity via induction of apoptosis	[279]
Prostate cancer	LNCaP, PC-3	LCA (25–75 µM)	LCA inhibits the proliferation of cancer cells and induces apoptosis	[273]
	PC-3, DU145	LCA (3–50 µM)	LCA decreases cell viability, induces apoptosis as well as induces endoplasmic reticulum stress, autophagy and mitochondrial dysfunction	[9]
	LNCaP, DU145	CDCA (50 µM)	Activation of FXR by CDCA inhibits cell proliferation and lipid accumulation via SREBF pathway	[270]
	LNCaP	CDCA (5 µM)	FXR activation by CDCA inhibits cell growth via upregulation of PTEN	[271]

*AP-1* activator protein-1, *BRCA1* breast cancer type 1 susceptibility protein, *CA* Cholic acid, *CAR* constitutive androstane receptor, *CDCA* Chenodeoxycholic acid, *C/EBP* CCAAT/enhancer-binding protein beta, *DCA* Deoxycholic acid, *EGF* epidermal growth factor, *EMT* epithelial–mesenchymal transition, *ER* estrogen receptor, *FXR* Farnesoid X receptor, *GADD153* growth arrest- and DNA damage-inducible gene 153, *GBC* Gallbladder cancer, *GCDC* Glycochenodeoxycholate, *GDC* Glycodeoxycholate, *HER2* human epidermal growth factor receptor 2, *LCA* Lithocholic acid, *MDM2* Mouse double minute 2, *MDM4* Double Minute 4, *MUC2* mucin 2, *NB* Neuroblastoma, *NDRG2* N-Myc downstream regulated gene 2, *NRF2* nuclear factor erythroid 2-related factor 2, *NFE2L2* PTEN, phosphatase and tensin homolog, *SOCS3* suppressor of cytokine signaling 3, *SREBF* sterol regulatory element-binding factor, *STAT3* signal transducer and activator of transcription 3, *TCA* Taurocholic acid, *TCDC* Taurochenodeoxycholate, *TDC* Taurodeoxycholate, *TGR5* G protein-coupled bile acid receptor 1, *VEGF* vascular endothelial growth factor, *VDR* vitamin D receptor

**Table 7:** Tumor promoter effects of bile acids in cancers

Cancer types	Cell lines	Concentration of bile acids	Effects of bile acids	Refs.
Breast cancer	4T1	DC (100 µM)	DC promotes survival of breast cancer cells by elevating *FLK-1* (KDR) and decreasing ceramide-mediated apoptosis of breast cancer progenitor cells	[341]
Cholangiocarcinoma	THLE-3	CDCA (100 µM) LCA (100 µM)	CDCA and LCA induce *Snail* and reduce E-cadherin expression and facilitate invasion and migration	[188]
	KMBC	TCDC, DC, GCDC (200 µM)	BAs participate in progression of cholangiosarcoma by activating EGFR and inducing *COX-2* expression via MAPK cascade	[342]
	human: HuCCT1, CCLP1, SG231, rat: BDE1, BDEspTDE_H10_	TCA (100 µM)	TCA promotes cholangiosarcoma cell invasion via activation of S1PR2. TCA induces invasive growth of cells, upregulate *COX2* expression and PGE2 production through S1PR2 receptor	[96] [95]
	RMCCA-1	TLCA	TLCA induces cell growth through muscarinic acetylcholine receptor (mAChR) and EGFR/ERK1/2 signaling pathways	[106]
Colon cancer / Colorectal carcinoma	HT29, SW620	LCA (30 µM)	LCA induces expression of urokinase-type plasminogen activator receptor (uPAR) and enhances cell invasiveness via ERK1/2 and AP-1 pathway	[343]
	H508, SNU-C4	LCT (300 µM)	LCT interacts with M3 muscarinic receptor and increases cell growth	[105]
	HCT-8/E11, SRC transformed PCmsrc cells	LCA, CDCA, DCA (10 µM)	BAs stimulate cellular invasion, which was dependent on several signaling pathways, such as RhoA, Rac1, PI3K, PKC, MAPK, COX2 and FXR receptor	[344]
	Normal human colonic epithelial cells (HCoEpiC)	LCA, DCA (100 µM)	BAs promote colon cancer by inducing cancer stemness in colonic epithelial cells via modulating CHRM3 and Wnt/β-catenin signaling	[238]
	CaCo-2	LCA (26.6 µM)	LCA increases cell invasion through promoting matrix metalloproteinase 2 (MMP-2) secretion	[345]
	HCT116, HT29	LCA (20 µM), DCA (150 µM)	BAs promote colon carcinogenesis via regulation of Nur77-mediated cell proliferation and apoptosis	[190]
	HCT116	LCA (30 µM)	LCA induces IL-8 expression by activating Erk1/2 MAPK and suppressing STAT3 Metformin inhibits LCA induced IL-8 upregulation in HCT116 cells by suppressing ROS production and NF-kB activity	[346] [347]
	SNU-C4, H508	GLCA, GDCA, (50–300 µM), DCA (300–1000 µM)	BAs induce colon cancer cell proliferation which is CHRM3-dependent and is mediated by transactivation of EGFR	[348]
	HCT116	DC (0.3–0.5 mM)	DC induces mitochondrial oxidative stress and activates NF-kB in cancer cells through multiple mechanisms involving NAD(P)H oxidase, Na^+^ /K^+^ -ATPase, CYP, Ca^2+^ and the terminal mitochondrial respiratory complex IV	[349]
	HT-29	DCA (250 µM)	DCA promotes colorectal tumorigenesis through activation of EGFR-MAPK pathway and induction of calcium signaling	[350]
	HT-29, Caco-2, HCA7, HCT116	DCA (300 µM)	DCA activates COX-2 signaling and mediates proliferation and invasiveness of colorectal epithelial cancer cells	[351]
	HCT-116, HCA-7	DCA (300 µM)	DCA activates EGFR, MAPK and STAT3 signaling and induces tumorigenicity. DCA-induced activation of cellular signaling is mediated by the TGR5	[226]
	SW480, LoVo	DCA (5–50 µM)	DCA activates β-catenin signaling and promotes colon cancer cell growth and invasiveness	[352]
	HCT116, DLD-1, SW620	DCA (100–200 µM)	DCA induces upregulation of *EPHA2* in colon cancer cells, which is due to activation of ERK 1/2 cascade, and is p53-independent	[353]
	Caco-2	DCA (20 µM)	DCA stimulates colon cancer-cell migration via PKC	[354]
	Caco-2, HT-29	DC < 20 µM > 100 µM	Low-dose (< 20 µM) DC stimulates colon cancer cell proliferation, while high dose (> 100 µM) induces apoptosis in colon cancer cells	[355]
	HCT116	DCA (250 µM)	DCA stimulates pro-apoptotic and anti-apoptotic signaling pathways; sensitivity to DCA induces apoptosis can be modulated by the ERK/MAP kinase	[356]
	HCT116	DCA (200 µM)	DCA suppresses p53 by stimulating proteasome-mediated degradation of p53. DCA suppression of p53 is mediated by stimulating the ERK signaling pathway	[357]
	HM3	DCA (200 µM)	DCA upregulates *MUC2* transcription via multiple pathways involving activation of EGFR/PKC/Ras/Raf-1/MEK1/ERK/CREB, PI3/Akt/IKKB/NF-κB and p38/MSK1/CREB while DCA induced *MUC2* transcription is inhibited by JNK/c-Jun/AP-1 pathway	[358]
	HT-29	DCA (50–500 µM)	DCA induces oxidative stress and upregulates Thioredoxin reductase (TR) mRNA	[359]
	HT-29	DCA (50–200 µM)	DCA activates anti-apoptotic effect of NF-κB and induces IL-8 expression	[360]
	murine model	/	DCA and tauro-β-muricholic acid have major role in promoting cancer stem cell proliferation	[361]
Endometrial cancer	Ishikawa	CDCA (5 µM)	CDCA enhances cyclin D1 expression and promotes cancer cell proliferation through TGR5-dependent CREB signaling activation	[362]
Gastric cancer	Normal human gastric epithelial cell: GES-1	CDCA, DCA (200 µM)	BAs upregulate *CDX2* and *MUC2* expression via activation of FXR/NF-κB signaling pathway	[176]
	Normal human gastric epithelial cell: GES-1 gastric carcinoma cell lines (AGS, MKN45, BGC823, AZ521, N87, KATO III, SGC7901)	DCA (200 µM)	DCA activates TGR5-ERK1/2 pathway following induction of *HNF4α* expression, which further promotes metaplasia markers expression through direct regulation of KLF4 and CDX2	[181]
	AGS	DCA (50 µM)	DCA activates ERK1/2, MAPK and causes a TGR5-dependent trans-phosphorylation of the EGFR	[182]
	MKN74, MKN45	TLCA, TDCA (100 µM)	Activation of TGR5 by BAs promotes EMT process	[183]
	MKN45, AGS	DCA (100 µM)	DCA enhances *COX-2* expression via CDX1 and SHP	[178]
	MKN28, MGC803, SGC7901	DCA, CDCA (100 µM)	BAs under acidic conditions increase *TERT* expression by activation of c-MYC transcription	[179]
Hepatocellular carcinoma (HCC)	HuH-7, Hep3B	CDCA (100 µM)	CDCA induces EMT phenotypes in HCC cells via FXR	[189]
	Huh7, Hep3B and mouse primary hepatocytes (MPH)	LCA (20 µM), DCA (150 µM)	BAs promote liver carcinogenesis via regulation of Nur77-mediated cell proliferation and apoptosis	[190]
	Huh-BAT, SNU-761, SNU-475	DCA (100 µM)	DCA induces ER stress accelerated apoptosis in NTCP-positive HCC cells under hypoxic conditions, while DCA induces COX-2-dependent *IL-8* overexpression in NTCP-negative human HCC cells mediated by NFκB	[191]
	SMMC7721, Huh7	GCDC (200 µM)	GCDC promotes HCC invasion and migration by AMPK/mTOR dependent autophagy activation	[363]
	HepG2, BeL-7402, Huh7	GCDA (100 µM)	GCDA contributes to the development of HCC and chemoresistance by inducing MCL1 phosphorylation at T163 via ERK1/2, which stabilizes MCL1 protein to enhance its antiapoptotic function	[364]
	HepG2, Bel7402, QGY7703, SMMC7721, Huh7	GCDA (100 µM)	GCDA induces survival and chemoresistance of liver cancer cells through activation of BCL-2 by phosphorylation	[365]
	LX2, Huh7	DCA (20–80 µM)	DCA causes HSC senescence by modulating malignant behavior of HCC	[192]
	HepG2	TCDCA (100 µM)	TCDCA promotes liver cancer via downregulation of the expression of tumor suppressor gene CEBPα	[366]
	Hep3B	LCA, CDCA (100 µM)	BAs increase cancer invasiveness in human hepatocellular carcinoma and cholangiocarcinoma through repressing E-cadherin and inducing Snail expression	[188]
Hypopharyngeal squamous cell carcinoma	FaDu cells	CA (100 µM), CDCA (100 µM), DCA (100 µM), LCA (20 µM)	BAs induce EMT markers *TGFβ1* and *MMP-9 *in vitro	[367]
Non-small cell lung cancer (NSCLC)	H1975, H1299, PC-9, A549	DCA (20–40 µM)	DCA increases cell migration and invasion through a TGR5-dependent way. TGR5 promotes NSCLC cell proliferaton and migration via JAK2/STAT3 pathway	[368]
Oesophageal adenocarcinoma (EAC) / Barett’s esophagus	HET-1A	DCA (300 µM), CDCA (300 µM), LCA (25 µM)	BAs activate the unfolded protein response and induce Golgi fragmentation via a src-kinase dependant mechanism contributing to cancer progression in the oesophagus	[369]
	SEG-1, BE3 CPC-A, CPC-C	CDCA (100–300 µM)	CDCA induces activation of IKKβ/TSC1/mTOR pathway leading to enhanced EAC cell proliferation	[370]
	OE-33, SK-GT-4	CDCA (100 µM)	CDCA stimulates the development of human esophageal cancer by promoting angiogenesis via the COX2 pathway	[371]
	HET-1A, QH	DCA (100–300 µM)	DCA promotes development of gastroesophageal reflux disease and Barrett’s oesophagus by modulating integrin-α_ν_ trafficking	[372]
	OE19, OE33	DCA (100, 300 µM)	DCA inhibits Notch signaling pathway with induction of *CDX2* gene expression contributing to the formation of Barrett’s oesophagus	[373]
	OE19	DCA (300 µM)	DCA shows carcinogenic effects via upregulation of *COX2*, *CDX2* and downregulation of DNA repair enzymes (*MUTYH, OGG1*)	[374]
	OE-19, OE-33	TCA (100 µM)	TCA promotes invasive growth of EAC cells via S1PR2	[165]
	OE19	DCA (50–300 µM)	DCA promotes the progression of EAC by inducing inflammation	[375]
	HET-1A, CP-A, CP-C, OE33	DCA (0.2 mM)	DCA increases *Beclin-1/BECN1* expression and autophagy but chronic exposure to BAs leads to decreased *Beclin-1/BECN1* expression and autophagy resistance	[376]
	BAR-T	DCA (250 µM)	DCA induces ROS/RNS production, which causes genotoxic injury, and simultaneously induces activation of the NF-κB pathway, which enables cells with DNA damage to resist apoptosis	[377]a
	OE33, KYSE-30	DCA (100–200 µM)	DCA is genotoxic to oesophageal cells at neutral and acid pH through the induction of ROS	[378]
		DCA ≥ 100 µM	DCA induces DNA damage and NF-kB activation (at doses of 100 uM and higher in oesophageal OE33 cells)	[379]
	SEG-1, SKGT-4, CP-A	CDCA, DCA (100 µM, 200 µM)	BAs induce CREB and AP-1-dependent *COX2* expression in Barrett’s oesophagus and EAC through ROS-mediated activation of PI3K/AKT and ERK1/2	[380]
	Het-1A, SEG-1, HKESC-1, HKESC-2	DCA (100–1000 µM)	DCA upregulates both intestinal differentiation factor *CDX2* and goblet cell-specific gene *MUC2* in normal esophageal and cancer cell lines suggesting the involvement of DCA in the pathogenesis of Barrett esophagus	[381]
	SEG-1 cells	DCA (50–300 µM)	DCA induces *MUC2* overexpression by activation of NF-kB transcription through a process involving PKC-dependent but not PKA, independent of activation of MAP kinase	[382]
	SKGT-4	DCA (300 µM)	DCA induces *COX2* expression via Erk1/2, p38-MAPK and AP-1-dependent mechanisms	[383]
	OE33 cells	DCA (250 µM)	DCA promotes the expression of *KLF4* and *OCT4* via IL-6/STAT3 signaling pathway. DCA has a malignancy-inducing effect on the transformation of EAC stem cells	[384]
	BAR-T, OA, FLO	TDCA (10^−11^ M)	TDCA induces cell proliferation through the upregulation of *NOX5*-S expression and ROS production mediated by activation of the TGR5 receptor	[164]
	OE33, FLO-1, Esc2	DCA (100 µM)	DCA enhances the aggressive phenotype of EAC cells with concomitant metabolic changes occurring via downregulation of *UCP2*	[385]
Pancreatic cancer	T3M4, HPAF, Capan-1	DCA, CDCA (5–100 µM)	BAs increase the tumorigenic potential of pancreatic cancer cells by inducing FXR/FAK/c-Jun axis to upregulate *MUC4* expression	[386]
	BxPC-3, AsPC-1, Capan-2	DCA (300 µM)	DCA activates EGFR, MAPK and STAT3 signaling and induces tumorigenicity. DCA-induced activation of cellular signaling is mediated by the TGR5	[226]

*AKT* Serine/Threonine Kinase 1, *AMPK* AMP-activated protein kinase, *AP-1* activator protein-1, *BA* bile acid, *Bcl-2* B-cell lymphoma 2, *Beclin-1/BECN1* Coiled-Coil Myosin-Like BCL2-Interacting Protein, *CDCA* chenodeoxycholic acid, *CDX1* Caudal Type Homeobox, *CDX2* Caudal Type Homeobox 2, *CEBPα* CCAAT/enhancer-binding protein alpha, *CHRM3* Muscarinic Acetylcholine Receptor M3, *COX2* cyclooxygenase-2, *CREB* cAMP response element-binding protein, *DC* Deoxycholate, *DCA* Deoxycholic acid, *EAC* Oesophageal adenocarcinoma, *EGFR* epithelial growth factor receptor, *EMT* epithelial-mesenchymal transition, *EPHA2* EPH Receptor A2, *ERK* extracellular signal-regulated kinase, *FAK/PTK2* focal adhesion kinase, *FLK1/KDR* Fetal liver kinase 1/Kinase Insert Domain receptor, *FXR* farnesoid X receptor, *GCDA* Glycochenodeoxycholate acid, *GCDC* Glycochenodeoxycholate, *GDCA* Glycodeoxycholic acid, *GLCA* Glycolithocholic acid, *HCC* hepatocellular carcinoma, *HNF4α* hepatocyte nuclear factor-4α, *HSC* hepatic stellate cells, *IKKβ/IKBKB* Inhibitor Of Nuclear Factor Kappa B Kinase Subunit Beta, *IL1* interleukin 1, *IL6* interleukin 6, *IL8/CXCL8* interleukin 8, *JAK2* Janus kinase 2, *JNK* c-Jun N-terminal kinase, *JUN* Jun Proto-oncogene, AP-1 Transcription Factor Subunit, *KLF4* Kruppel Like Factor 4, *LCA* Lithocholic acid, *LCT* Lithocholyltaurine, *mAChR* muscarinic acetylcholine receptor, *MAPK/MEK* mitogen-activated protein kinase, *MCL1* Induced myeloid leukemia cell differentiation protein, *MMP2* matrix metalloproteinase 2, *MMP9* matrix metalloproteinase-9, *MSK1/RPS6KA5* Nuclear Mitogen- And Stress-Activated Protein Kinase 1, *mTOR* mammalian/mechanistic target of Rapamycin, *MUC2* Mucin 2, *MUC4* Mucin 4, *MUTYH* MutY DNA Glycosylase, *MYC* Myc Proto-Oncogene Protein, *NF-κB* nuclear factor κB, *NOX5* NADPH Oxidase 5, *NR4A1/Nur77/TR3/NGFIB* Nuclear receptor subfamily 4 group A member 1, *NSCLC* non-small cell lung cancer, *NTCP/SLC10A1* sodium/taurocholate cotransporting polypeptide, *OCT4/POU5F1* Octamer-Binding Transcription Factor, *OGG1* 8-Oxoguanine DNA Glycosylase, *p38/MAPK14* p38 MAP Kinase, *PGE2* prostaglandin E2, *PI3K* Phosphatidylinositol 3-kinase, *PKA* protein kinase A, *PKC* protein kinase C, *Rac1* Rac Family Small GTPase 1, *Raf1* Proto-Oncogene, Serine/Threonine Kinase, *RhoA* Ras Homolog Family Member A, *RNS* reactive nitrogen species, *ROS* reactive oxygen species, *S1PR2* sphingosine 1-phosphate receptor 2, *SHP* Small heterodimer partner, *STAT* signal transducer and activator of transcription, *TCA* Taurocholic acid, *TCDC* Taurochenodeoxycholate, *TCDCA* Taurochenodeoxycholic acid, *TDCA* Taurodeoxycholic acid, *TERT* Telomerase Reverse Transcriptase, *TGF-β1* Transforming growth factor β-1, *TGR5/GPBAR1* G-protein-coupled bile acid receptor/Takeda-G-protein-receptor-5, *TLCA* Taurolithocholic acid, *TSC1* TSC Complex Subunit 1, *TXNRD1* Thioredoxin reductase 1, *UCP2* uncoupling protein-2, *uPAR/PLAUR* urokinase-type plasminogen activator receptor, *WNT* wingless-type MMTV integration site family

### Oesophageal carcinoma

The development of Barrett’s esophagus (BE) and its progression to oesophageal adenocarcinoma (EAC) are linked to gastroesophageal reflux disease (GERD). Conjugated BAs, mainly taurocholic acid (TCA) and glycocholic acid (GCA) are the main BA constituents in GERD refluxate [162]. Conjugated BA levels in the refluxate from patients with advanced BE or EAC are significantly higher than from patients with benign BE [163]. Conjugated BAs, as TCA or taurodeoxycholic acid (TDCA), promote EAC progression [164, 165] (Table 7). Unconjugated BAs, including DCA and CDCA, induce oxidative stress, DNA damage and inflammation contributing to EAC carcinogenesis, while UDCA protects against DCA-induced injury (Tables 5 and 7).

Apparently, numerous BA receptors as TGR5, S1PR2, FXR and VDR are activated in EAC cells in response to BAs in the refluxate [164–167]. In good agreement with that, the inhibition of the FXR receptor suppresses tumor cell viability in vitro and reduced tumor formation in nude mouse xenografts [168]. Furthermore, TGR5 is highly expressed in the EAC and precancerous lesions and is associated with worse overall survival [169] suggesting that these observations can be translated to the human situation.

Acidic bile acids bring about oxidative stress, TDCA can induce NADPH Oxidase 5 (NOX5) through TGR5 [164]. Furthermore, bile acids can induce inflammation through FXR activation [170] and the EGFR–STAT3 (signal transducer and activator of transcription 3)—Apurinic/Apyrimidinic Endodeoxyribonuclease 1 (APE1) pathway [171]. Acidic bile salts can also induce epithelial–mesenchymal transition (EMT) through vascular endothelial growth factor (VEGF) signaling in Barrett's cells [172]. Interestingly, the activation of the EGFR-DNA-PKs (DNA-dependent protein kinase) pathway by insulin-like growth factor binding protein 2 (IGFBP2) protects EAC cells against acidic bile salt-induced DNA damage [173].

### Gastric cancer

Carcinogenesis in gastric cancer is a sequential process that includes chronic superficial gastritis, intestinal metaplasia (IM), atrophic gastritis, intramucosal carcinoma, dysplasia and invasive neoplasia [174]. IM is considered a risk factor for gastric tumorigenesis. The concentrations of BAs in gastric juice positively correlate with the degree of intestinal metaplasia [175] and BAs serve a critical multipronged role in the induction of intestinal metaplasia. BAs can enhance caudal-related homeobox family 2 (CDX2) and mucin 2 (MUC2) expression via FXR/NF-κB signaling [176, 177] and cyclooxygenase-2 (COX-2) expression via induction of SHP [178], all promoting gastric intestinal metaplasia. Acidic bile salts can induce telomerase activity in a c-Myc-dependent fashion [179, 180], while DCA can induce the metaplastic phenotype of gastric cancer cells [181] (see Tables 6 and 7). TGR5 is a key factor in BA-induced gastric metaplasia via HNF4α [181], EGFR and mitogen-activated protein kinase (MAPK) [182] activation and promotes EMT in gastric carcinoma cells [183]. TGR5 is overexpressed in gastrointestinal adenocarcinomas, and moderate to strong TGR5 staining is associated with decreased patient survival [184]. Nevertheless, there anticarcinogenic effects of bile acids in gastric cancer, as UDCA (Table 5) or DCA in supraphysiological concentrations [185, 186] or 23(S)-mCDCA [187].

### Hepatocellular carcinoma (HCC)

Several studies have shown that more hydrophobic BAs as LCA, DCA and CDCA, are the main promoters of liver cancer and can contribute to the development of HCC (see in Table 7) [188–192]. Nevertheless, CDCA (> 100 µM) [193, 194], UDCA and Tauroursodeoxycholic acid (TUDCA) inhibit HCC cell growth and induce apoptosis [195–199] (see in Tables 5 and 6). Deregulation of BA homeostasis marked by the expression of hepatic BA transporters (BSEP, OSTα/β, MRP2, MDR2-3, NTCP) is diminished leading to increased hepatic BA sequestration and inflammation and reduced FXR signaling [200–203] in liver cirrhosis and nonalcoholic steatohepatitis that are risk factors for the development of HCC. In good agreement with that, metabolomics identified long-term elevated serum BAs in HCC patients [204] and children (< 5 years of age) with bile salt export pump deficiency developed HCC [205].

FXR activity is a major inhibitor of HCC carcinogenesis. Whole-body FXR-deficient mice spontaneously develop liver tumors [206, 207] in which the activation of the Wnt/β-catenin signaling pathway and oxidative stress were identified as the major drivers [208–210]. Nevertheless, liver-specific FXR deficiency in mice does not induce spontaneous liver tumorigenesis, but may only serve as a tumor initiator [211]. Due to their amphipathic nature, BAs can disrupt the plasma membrane and activate protein kinase C (PKC) and phospholipase A2 (PLA2) inducing the p38-MAPK-p53-NFκB pathway [212, 213]. Inflammation can suppress FXR activity that contributes to bile acid accumulation and carcinogenesis [185, 193, 194, 214].

Interestingly, senescence-associated secretory phenotype has crucial role in promoting obesity-associated HCC development in mice. Administration of high-fat diet to mice induces alterations in the gut microbiota and increases the levels of DCA. Increased DCA level promotes SASP phenotype in hepatic stellate cells (HSCs), which in turn secretes various tumor-promoting factors in the liver, thus facilitating HCC development in mice exposed to chemical carcinogen [6]. SHP has a pleiotropic role in HCC, regulates cell proliferation [215], apoptosis [216], epigenetic changes [217] and inflammation [200, 218], which are associated with the antitumor role of SHP in the development of liver cancer.

### Pancreatic adenocarcinoma

BAs are involved in the induction and development of pancreatic adenocarcinoma at multiple stages. Gallstone formation can block bile flow and, therefore, can induce and sustain pancreatitis [219], a risk factor for pancreatic adenocarcinoma [220–222]. In fact, several BA species showed a drastic increase in pancreatic adenocarcinoma patients [223]. Treatment of pre-malignant pancreas ductal cells with bile induced carcinogenic transformation [224, 225]. In pancreatic adenocarcinoma cells BAs decrease susceptibility to apoptosis, boost cell cycle progression, the expression of inflammatory mediators and cellular movement, and, in high concentrations, may perturb biomembranes (Table 7) [220, 226]. UDCA, similar to its previously discussed beneficial properties, prevents EMT in pancreatic adenocarcinoma cell lines and, therefore, has antineoplastic properties (Table 5) [227].

### Colorectal carcinoma (CRC)

The western diet has tumor promoting activity associated with elevated concentrations of colonic BA (mainly LCA and DCA) and increased fecal BA levels, as detected in samples from CRC patients [228]. In animals, a high-fat diet stimulates bile discharge and results in elevated BA levels in the colon [229]. Moreover, cholecystectomy, through prolonging BA exposure of the intestinal mucosa, has been suggested as a risk factor for the development of CRC [230].

BAs induce genetic instability marked by genomic instability and DNA damage via oxidative stress, defects in mitotic checkpoints, cell cycle arrest, improper chromosome alignment and multipolar division [231, 232]. Genomic instability caused by BAs is coupled with apoptosis resistance due to the degradation of p53 and the inhibition of caspase-3 activity [233]. Furthermore, secondary BAs perturb cell membranes and modulate signaling cascades [234, 235]. These all lead to colonic cell hyperproliferation, survival and invasion [236, 237].

The disruptive effect of BAs on colon epithelium evokes a compensatory cell renewal mechanism by inducing colonic epithelial cells to become cancer stem cells (CSCs) through β-catenin signaling (Table 7) [238]. In the CRC rodent model, both LCA and DCA have tumor promoter role on colonic crypt cells in the early stages of colon carcinogenesis [239]; however, it is important to note that BAs are suggested as tumor promoters, but not as mutagenic agents, since they can not induce tumor formation without a carcinogen/mutagen or a genetic alteration [240, 241]. It should be noted that DCA in low concentrations (0.05–0.3 mM) inhibit colonic cell proliferation via cell cycle block and apoptosis pathways (Table 6) [242].

UDCA can reduce the concentration of toxic BA in stool and blood [243] and has shown to protect against CRC by inhibiting CSC and CRC cell formation and proliferation [244, 245], oncogenic signaling pathways [246], as well as, inducing tumor surveillance [247] (Table 5). Moreover, UDCA can reduces CRC recurrence [248], as well as the risk to develop CRC in patients with pre-cancerous conditions, as colitis [249] or primary biliary cirrhosis [250].

Sustained inflammation was implicated in the pathogenesis of colorectal cancer due to barrier breach, and bacterial translocation leading to inflammation and neoplastic transformation of colonic epithelial cells [251–253]. TGR5 activation by UDCA and LCA may also exert anti-inflammatory responses through TLR4 activation or by reducing pro-inflammatory cytokine production in the colon that can decrease the frequency of developing CRC [254]. BAs can change the gut microbial community [255, 256], suggesting that BAs may also interfere with bacterial translocation.

### Breast cancer

The BAs in the breast are of gut origin [257, 258]. Hepatic production of BA is reduced in breast cancer patients as marked by decreasing levels of serum and fecal BAs [7, 259]. Furthermore, bacterial conversion of BAs to secondary BAs is also suppressed, which is the most dominant in in situ and stage I patients [7]. The serum bile acid composition of breast cancer and benign breast disease patients is different; specifically, breast cancer patients had higher serum chenodeoxycholic acid levels and lower dihydroxy tauro-conjugated BA (Tdi-1) and sulfated dihydroxy glyco-conjugated bile acids (Gdi-S-1) [260]. Total fecal bile acid levels are lower in breast cancer patients as compared to controls [259]. LCA concentrations in the breast can be higher than the serum levels [261] (Table 6). Reports showed increased DCA levels in the serum [262] and the breast cyst fluid [263] of breast cancer patients.

LCA is an inhibitor of breast cancer cell proliferation (Table 6) [7, 258, 264]. However, the reports on DCA and UDCA are contradictory [7, 258, 262–264] in physiological concentrations, LCA tunes cancer cell metabolism towards a more oxidative state (through AMP-activated protein kinase (AMPK), PGC-1β and NRF1/NFE2L1) and induces mild oxidative stress through reducing NRF2 (nuclear factor erythroid 2-related factor 2, NFE2L2) expression and inducing Inducible nitric oxide synthase (iNOS) that reverts EMT, reduces VEGF expression, induces antitumor immunity and changes to cancer metabolism that culminates in reduced metastasis formation [7, 11]. In supraphysiological concentrations (> 1 µM) LCA inhibits fatty acid biosynthesis [10] and induces cell death [8–10, 265, 266]. LCA does not exert antiproliferative effects in its tissue reference concentrations on non-transformed primary fibroblasts [7]. LCA exerts its antineoplastic effects through the TGR5 [7] (Table 6).

CDCA in supraphysiological concentrations induces MDRs through FXR [265] and modulates estrogen and progesterone receptor-mediated gene transcription [267]. Furthermore, CDCA inhibits tamoxifen-resistant breast cancer cell proliferation through the activation of the FXR receptor [268] (Table 6). In contrast to that, a report by Journe and colleagues [269] showed that FXR activation has a positive correlation with estrogen receptor expression and luminal characteristics, as well as supported cancer cell proliferation.

### Prostate cancer

Among the BAs LCA, UDCA and CDCA exerted antiproliferative effects in prostate cancer. Activation of FXR by CDCA inhibits proliferation of prostate cancer cells, reduces lipid anabolism via inhibiting Sterol Regulatory Element Binding Transcription Factor 1 (SREBF1) [270] and induces the expression of the tumor suppressor phosphatase and tensin homolog (PTEN) [271] (Table 6). Interestingly, FXR signaling also controls androgen metabolism in prostate cancer cells, its activation reduces the expression of UDP-glucuronosyltransferase (UGT) 2B15 and UGT2B17 within cells and causes a reduction of androgen glucuronidation [272]. Similar to CDCA, LCA has antiproliferative effects in prostate cancer and induces apoptosis, endoplasmic reticulum stress, autophagy and mitochondrial dysfunction [9, 273] (see Table 6). UDCA induces death receptor-mediated apoptosis in human prostate cancer cells [274] (Table 5).

### Ovarian cancer

In the serum of ovarian cancer patients, 3b-hydroxy-5-cholenoic acid, GUDCA, DCA and TCDCA levels decreased [275, 276]; importantly, taurochenodeoxycholic acid levels decreased in early-stage epithelial ovarian cancer [276]. Zhou and colleagues have shown that sulfolithocholylglycine and TCA showed changes in the serum of ovarian cancer patients [277]. Changes to the BA pool are so characteristic that Guan and colleagues suggested [278] a set of 12 BAs, including glycolithocholic acid, to be used as markers to separate healthy controls from ovarian cancer patients.

The available studies assessed the effects of BAs at supraphysiological concentrations. These concentrations of BAs are cytotoxic and induce apoptosis likely due to changes to membrane damage [279, 280] that is unlikely at physiological concentrations of BAs [7]. DCA can modulate the expression of breast cancer type 1 susceptibility protein (BRCA1) and the estrogen receptor and, through these, can control drug sensitivity of ovarian cancer cells (Table 6) [281]. Furthermore, cholylglycinate interferes with the transport of cisplatin [282] and TCDC sensitizes ovarian carcinoma cells to doxorubicin and Mitomycin [280].

LXR [283–285], PXR [286], VDR [287–296] or CAR [297, 298] activation was shown to exert protective features against ovarian cancer, similar to BA-elicited effects suggesting that BAs may have a more profound role in protecting against ovarian cancer. These protective effects involved the suppression of proliferation [283, 284, 286], invasion [291], EMT [288], de novo fatty acid biosynthesis [295], the proportions of the cancer stem cell population [289], and the improvement of the efficacy of chemotherapy [285, 297, 298] culminating in better patient survival [292, 293]. Conflicting with these observation on report provided evidence that under certain conditions PXR may support proliferation [299]. BAs can influence the expression and the activity of multiple PARP enzymes [300]; therefore, it is likely that BAs could modulate the efficacy of PARP inhibition that is a novel modality in the chemotherapy of ovarian cancer.

## Conclusions

Primary and secondary BAs are long-standing players in carcinogenesis. Although these molecules were considered as initiators of neoplasias, recent advances have shown that the pro- or anticarcinogenic activity of BAs varies among neoplasias [301], most probably due to differences in the expression of BA receptors, transporters and cell-specific differences in the outcome of receptor activation. Key pathways activated in neoplasias by BAs are regulated by nuclear receptors, FXR, CAR, SHP, PXR, LXR and VDR and other membrane receptors such as S1PR2, TGR5, CHRM2 and CHRM3. They activate numerous downstream signaling pathways such as EGFR, STAT3, MAPK, HNF4α, NF-κB, TLR4, SOCS3 and β-catenin just to name some. Furthermore, BAs regulate all aspects of tumor development and progression, the EMT, invasion, metabolism, apoptosis, proliferation, senescence, immune environment and response to chemotherapy.

The effect of BAs on neoplasias also depends on the concentrations used in the studies. While in certain models BAs in low concentration have anti-cancer effects, in superphysiological concentrations BAs have pro-cancer effects. This phenomenon is related to their amphipathic structure and the activation of additional off-target pathways not tiggered at physiological concentration. At high concentrations, BAs may perturb membranes and activate signaling pathways that sense disturbance of membranes, such as PLA2 and PKC. At high concentrations, they are also toxic and activate the detoxifying pathways, which regulate the activity of transporters of steroid hormones and chemotherapeutics. Therefore, we would urge the community to carry out studies where the concentrations of BAs correspond to the reference concentrations established for the tissue or, as a proxy, to the serum reference concentrations. As a continuation of that, in the case of UDCA the therapeutic serum concentrations can also be used as a guide. These data are summarized in Table 1. Such studies would be invaluable to understand the (patho)physiological roles of BAs and would give a good frame for the therapeutic applicability.

Along the same lines, it is apparent that BAs can be considered as possible treatment options in certain cancers. Foremost, UDCA, that is a therapeutically available drug, has beneficial effects in multiple neoplasias (e.g. [227, 248, 302], Table 5) pointing towards the possibility for repurposing UDCA. The picture for other BAs is hazier due to frequent contradictions making it hard to outline applicability. However, before the application of BAs in neoplasias we would need to decipher the cross-talk between BAs and drug metabolism, the effect on drug efficacy and drug availability, and discover the possible adverse effects of BAs, that is currently largely missing. Moreover, it is tempting to consider the manipulation of the intestinal microbiome to affect the levels of selected secondary bile acids in humans*.* Finally, the modulators of BA receptors should be considered as therapeutic options as well. Given the emerging evidence on the potential anti-cancer effects of BAs, further studies are vital in order to develop novel therapeutic strategies using BAs.

## Search strategy and selection criteria

References to this review were identified through the prior knowledge of the authors that was complemented by systematic search of PubMed by using the combinations “Prostate cancer AND (bile acid)”, “Gastric cancer AND (bile acid)”, “Hepatocellular carcinoma AND (bile acid)”, “Oesophageal cancer AND (bile acid)”, “(bile acid) receptors AND cancer”, “(bile acid) receptors AND prostate cancer”, “(bile acid) receptors AND gastric cancer”, “(bile acid) receptors AND hepatocellular carcinoma”, “(bile acid) receptors AND oesophageal cancer”, "(bile acid) AND ABC AND transporter", "(bile acid) AND SLC AND transporter", "(bile acid) AND SLCO AND transporter", "(bile acid) AND transport AND review", “Farnesoid X receptor (FXR) AND the cancer types assessed in the study”, “Pregnane X receptor (PXR) AND the cancer types assessed in the study”, “Constitutive androstane receptor (CAR) AND the cancer types assessed in the study”, “Vitamin D receptor (VDR) AND the cancer types assessed in the study” “Liver X receptor (LXR) AND the cancer types assessed in the study”, “Small heterodimer partner (SHP) AND the cancer types assessed in the study”. Articles published in English were included with no restriction on publication date. All references were checked at Pub Peer, two papers were flagged ([215] and [156]), but when reviewing the reports we decided that the issues raised do not impact on the main message and kept the references.

## Data Availability

Not applicable.

## Abbreviations

AKT: Serine/threonine kinase 1
AMPK: AMP-activated protein kinase
AP-1: Activator protein-1
APE1: Apurinic/apyrimidinic endodeoxyribonuclease 1
ATG5: Autophagy related 5
BA: Bile acids
Bai: Bile acid inducible operon
Bax: Bcl-2-associated X protein
Bcl-2: B-cell lymphoma 2
BE: Barrett’s esophagus
Beclin-1/BECN1: Coiled-coil myosin-like BCL2-interacting protein
BIRC7/Livin: Baculoviral IAP repeat-containing protein 7
BSEP/ABCB11: ATP-dependent cassette transporter
BSH: Bile salt hydrolases
BRCA1: Breast cancer type 1 susceptibility protein
CA: Cholic acid
cAMP: Cyclic adenosine monophosphate
CAR/NR1H3: Constitutive androstane receptor
CDCA: Chenodeoxycholic acid
CDX1/2: Caudal type homeobox 1/2
C/EBPα: CCAAT/enhancer-binding protein alpha
CHRM2/3: Muscarinic receptor 2/3
c-Myc: Myc-related translation/localization regulatory factor
COX2: Cyclooxygenase-2
CRC: Colorectal carcinoma
CREB: CAMP response element-binding protein
CSC: Cancer stem cells
CYP: Cytochrome P450
CYP7A1: Cholesterol 7α-hydroxylase
CYP7B1: 25-Hydroxycholesterol 7α-hydroxylase
CYP8B1: Sterol 12α-hydroxylase
CYP27A1: Sterol 27-hydroxylase
CYP3A4: Cytochrome P450 family 3 subfamily
DC: Deoxycholate
DCA: Deoxycholic acid
Dlc1: Deleted in Liver Cancer 1
DNA-PK: DNA-dependent protein kinase
DR5: Death receptor 5
EAC: Oesophageal adenocarcinoma
EGF: Epidermal growth factor
EGFR: Epithelial growth factor receptor
EMT: Epithelial–mesenchymal transition
EPHA2: EPH Receptor A2
ER: Estrogen receptor
ERK: Extracellular signal-regulated kinase
FAK/PTK2: Focal adhesion kinase
FAS: Fas Cell Surface Death Receptor
FGF19: Fibroblast growth factor 19
FGF15: Fibroblast growth factor 15
FGFR4: Fibroblast growth factor receptor 4
FLK1/KDR: Fetal liver kinase 1/Kinase Insert Domain receptor
FXR/ NR1H4: Farnesoid X receptor
FXREs: FXR response elements
GADD153: Growth arrest- and DNA damage-inducible gene 153
GBC: Gallbladder cancer
GERD: Gastroesophageal reflux disease
GCA: Glycocholic acid
GCDCA: Glycochenodeoxycholic acid
GCDA: Glycochenodeoxycholate acid
GCDC: Glycochenodeoxycholate
GDC: Glycodeoxycholate
GDCA: Glycodeoxycholic acid
GLCA: Glycolithocholic acid
GPBAR1/TGR5: G-protein-coupled bile acid receptor/Takeda-G-protein-receptor-5
GUDCA: Glycoursodeoxycholic acid
HCC: Hepatocellular carcinoma
HDCA: Hyodeoxycholic acid
HER2: Human epidermal growth factor receptor 2
HNF4α: Hepatocyte nuclear factor-4α
HSC: Hepatic stellate cells
I-BABP: Intestinal BA-binding protein
IGFBP2: Insulin-like growth factor binding protein 2
IKKβ/IKBKB: Inhibitor Of Nuclear Factor Kappa B Kinase Subunit Beta
IL1: Interleukin 1
IL6: Interleukin 6
IL8/CXCL8: Interleukin 8
iNOS: Inducible nitric oxide synthase
JAK2: Janus kinase 2
JNK: C-Jun N-terminal kinase
JUN: Jun Proto-Oncogene AP-1 Transcription Factor Subunit
KLF4: Kruppel Like Factor 4
LBD: Ligand-binding domain
LCA: Lithocholic acid
LCT: Lithocholyltaurine
LOD: Limit of detection
LRH-1/NR5A2: Liver receptor homolog-1
LXRα/β/NR1H3-2: Liver X receptor
mAChR: Muscarinic acetylcholine receptor
MAPK/MEK: Mitogen-activated protein kinase
MCA: Muricholic acid
MCL1: Induced myeloid leukemia cell differentiation protein
MDM2: Mouse double minute 2
MDM4: Double Minute 4
MDR1/ ABCB1: Multidrug resistance protein 1
MMP2: Matrix metalloproteinase 2
MMP9: Matrix metalloproteinase 9
MRP2/ABCC2: Multidrug resistance-associated protein 2
MRP3/ABCC3: Multidrug resistance-associated protein 3
MRP4/ABCC4: Multidrug resistance-associated protein 4
MSK1/RPS6KA5: Nuclear mitogen- and stress-activated protein kinase 1
mTOR: Mammalian target of rapamycin
mTORC1: Mammalian target of rapamycin complex 1
MUC2: Mucin 2
MUC4: Mucin 4
MUTYH: MutY DNA Glycosylase
MYC: Myc proto-oncogene protein
NB: Neuroblastoma
NDRG2: N-Myc downstream regulated gene 2
ND: Not detected
NF-κB: Nuclear factor κappa-light-chain-enhancer of activated B cells
NOX5: NADPH Oxidase 5
NR: Nuclear receptor
NRF2/NFE2L2: Nuclear factor erythroid 2-related factor 2
NR4A1/Nur77/TR3/NGFIB: Nuclear receptor subfamily 4 group A member 1
NSCLC: Non-small cell lung cancer
NTCP/SLC10A1: Sodium/taurocholate cotransporting polypeptide
OATP1A2/SLCO1A2: Solute carrier organic anion transporter family member 1A2
OATP1B/SLCO1B: Solute carrier organic anion transporter family
OATP2: Organic anion-transporting polypeptide
OCT4/POU5F1: Octamer-binding transcription factor
OGG1: 8-Oxoguanine DNA glycosylase
PGC-1α: Peroxisome proliferator-activated receptor gamma coactivator 1 alpha
PGE2: Prostaglandin E2
PI3K: Phosphatidylinositol 3-kinase
PKA: Protein kinase A
PKC: Protein kinase C
PLA2: Phospholipase A2
Prx2: Peroxiredoxin II
PXR/ NR1H2: Pregnane X receptor
PTEN: Phosphatase and tensin homolog
p38/MAPK14: P38 MAP kinase
Rac1: Rac family small GTPase 1
Raf1: Proto-oncogene, serine/threonine kinase
RhoA: Ras homolog family member A
RNS: Reactive nitrogen species
ROS: Reactive oxygen species
RXR: Retinoid X receptor
S1PR2: Sphingosine-1-phosphate receptor 2
SHP/ NR5O2: Small heterodimer partner
SLC10A1/NTCP: Solute carrier family 10
SLC10A2/ASBT: Sodium-dependent bile acid transporter
SLC51A/B or OSTα/β: Solute carrier family members
SRC-1/NC0A1: Steroid receptor coactivator 1
Smac: Second mitochondria-derived activator of caspase
SOCS3: Suppressor of cytokine signaling 3
SphK2: Sphingosine kinase 2
SRC-1/NC0A1: Steroid receptor coactivator 1
SREBF: Sterol regulatory element-binding factor
STAT3: Signal transducer and activator of transcription 3
SULT: Sulfotransferase
TCA: Taurocholic acid
TCDC: Taurochenodeoxycholate
TCDCA: Taurochenodeoxycholic acid
TDC: Taurodeoxycholate
TDCA: Taurodeoxycholic acid
TERT: Telomerase Reverse Transcriptase
TGF-β1: Transforming growth factor β-1
TLC: Taurolithocholate
TLCA: Taurolithocholic acid
TLR4: Toll-Like Receptor 4
TSC1: TSC Complex Subunit 1
TUDCA: Tauroursodeoxycholic acid
UCP2: Uncoupling protein-2
UDCA: Ursodeoxycholic acid
UGT: UDP-glucuronosyl-transferase
UGT2B4: Uridine 5′-diphosphate-glucuronosyltransferase 2B4
uPAR/PLAUR: Urokinase-type plasminogen activator receptor
VDR/NR1H1: Vitamin D receptor
VEGF: Vascular endothelial growth factor
WNT: Wingless-type MMTV integration site family

## References

[1] Stieger B (2003). Biliary cholesterol secretion: more lessons from plants?. J Hepatol.

[2] Pellicciari R, Gioiello A, Costantino G (2006). Potential therapeutic applications of farnesoid X receptor (FXR) modulators. Expert Opin Ther Pat.

[3] Cai X, Young GM, Xie W (2021). The xenobiotic receptors PXR and CAR in liver physiology, an update. Biochim Biophys Acta Mol Basis Dis.

[4] Sipos A, Ujlaki G, Mikó E, Maka E, Szabó J, Uray K, Krasznai Z, Bai P (2021). The role of the microbiome in ovarian cancer: mechanistic insights into oncobiosis and to bacterial metabolite signaling. Mol Med.

[5] Kiss B, Mikó E, Sebő É, Toth J, Ujlaki G, Szabó J, Uray K, Bai P, Árkosy P (2020). Oncobiosis and microbial metabolite signaling in pancreatic adenocarcinoma. Cancers (Basel).

[6] Yoshimoto S, Loo TM, Atarashi K, Kanda H, Sato S, Oyadomari S, Iwakura Y, Oshima K, Morita H, Hattori M, Honda K, Ishikawa Y, Hara E, Ohtani N (2013). Obesity-induced gut microbial metabolite promotes liver cancer through senescence secretome. Nature.

[7] Miko E, Vida A, Kovacs T, Ujlaki G, Trencsenyi G, Marton J, Sari Z, Kovacs P, Boratko A, Hujber Z, Csonka T, Antal-Szalmas P, Watanabe M, Gombos I, Csoka B, Kiss B, Vigh L, Szabo J, Mehes G, Sebestyen A, Goedert JJ, Bai P (1859). Lithocholic acid, a bacterial metabolite reduces breast cancer cell proliferation and aggressiveness. Biochim Biophys Acta Bioenerg.

[8] Goldberg AA, Beach A, Davies GF, Harkness TA, Leblanc A, Titorenko VI (2011). Lithocholic bile acid selectively kills neuroblastoma cells, while sparing normal neuronal cells. Oncotarget.

[9] Gafar AA, Draz HM, Goldberg AA, Bashandy MA, Bakry S, Khalifa MA, AbuShair W, Titorenko VI, Sanderson JT (2016). Lithocholic acid induces endoplasmic reticulum stress, autophagy and mitochondrial dysfunction in human prostate cancer cells. PeerJ.

[10] Luu TH, Bard JM, Carbonnelle D, Chaillou C, Huvelin JM, Bobin-Dubigeon C, Nazih H (2018). Lithocholic bile acid inhibits lipogenesis and induces apoptosis in breast cancer cells. Cell Oncol.

[11] Kovács P, Csonka T, Kovács T, Sári Z, Ujlaki G, Sipos A, Karányi Z, Szeőcs D, Hegedűs C, Uray K, Jankó L, Kiss M, Kiss B, Laoui D, Virág L, Méhes G, Bai P, Mikó E (2019). Lithocholic acid, a metabolite of the microbiome, increases oxidative stress in breast cancer. Cancers (Basel).

[12] Rezen T, Rozman D, Pascussi JM, Monostory K (1814). Interplay between cholesterol and drug metabolism. Biochim Biophys Acta Proteins Proteom.

[13] Hafner M, Rezen T, Rozman D (2011). Regulation of hepatic cytochromes p450 by lipids and cholesterol. Curr Drug Metab.

[14] Honda A, Miyazaki T, Iwamoto J, Hirayama T, Morishita Y, Monma T, Ueda H, Mizuno S, Sugiyama F, Takahashi S, Ikegami T (2020). Regulation of bile acid metabolism in mouse models with hydrophobic bile acid composition. J Lipid Res.

[15] Lorbek G, Lewinska M, Rozman D (2012). Cytochrome P450s in the synthesis of cholesterol and bile acids–from mouse models to human diseases. FEBS J.

[16] Monte MJ, Marin JJ, Antelo A, Vazquez-Tato J (2009). Bile acids: chemistry, physiology, and pathophysiology. World J Gastroenterol.

[17] MahmoudianDehkordi S, Arnold M, Nho K, Ahmad S, Jia W, Xie G, Louie G, Kueider-Paisley A, Moseley MA, Thompson JW, John Williams L, Tenenbaum JD, Blach C, Baillie R, Han X, Bhattacharyya S, Toledo JB, Schafferer S, Klein S, Koal T, Risacher SL, Kling MA, Motsinger-Reif A, Rotroff DM, Jack J, Hankemeier T, Bennett DA, De Jager PL, Trojanowski JQ, Shaw LM, Weiner MW, Doraiswamy PM, van Duijn CM, Saykin AJ, Kastenmuller G, Kaddurah-Daouk R (2019). Altered bile acid profile associates with cognitive impairment in Alzheimer's disease-An emerging role for gut microbiome. Alzheimers Dement.

[18] Miko E, Kovacs T, Sebo E, Toth J, Csonka T, Ujlaki G, Sipos A, Szabo J, Mehes G, Bai P (2019). Microbiome-microbial metabolome-cancer cell interactions in breast cancer-familiar, but unexplored. Cells.

[19] Sarin SK, Pande A, Schnabl B (2019). Microbiome as a therapeutic target in alcohol-related liver disease. J Hepatol.

[20] Watanabe M, Houten SM, Mataki C, Christoffolete MA, Kim BW, Sato H, Messaddeq N, Harney JW, Ezaki O, Kodama T, Schoonjans K, Bianco AC, Auwerx J (2006). Bile acids induce energy expenditure by promoting intracellular thyroid hormone activation. Nature.

[21] Hofmann AF, Mysels KJ (1987). Bile salts as biological surfactants. Colloids Surf.

[22] Bertani B, Ruiz N (2018). Function and biogenesis of lipopolysaccharides. EcoSal Plus.

[23] Ridlon JM, Harris SC, Bhowmik S, Kang DJ, Hylemon PB (2016). Consequences of bile salt biotransformations by intestinal bacteria. Gut Microbes.

[24] Ridlon JM, Kang DJ, Hylemon PB (2006). Bile salt biotransformations by human intestinal bacteria. J Lipid Res.

[25] Kuang J, Zheng X, Huang F, Wang S, Li M, Zhao M, Sang C, Ge K, Li Y, Li J, Rajani C, Ma X, Zhou S, Zhao A, Jia W (2020). Anti-adipogenic effect of theabrownin is mediated by bile acid alternative synthesis via gut microbiota remodeling. Metabolites.

[26] Yesair DW, Himmelfarb P (1970). Hydrolysis of conjugated bile acids by cell-free extracts from aerobic bacteria. Appl Microbiol.

[27] Aries V, Hill MJ (1970). Degradation of steroids by intestinal bacteria. I Deconjugation of bile salts. Biochim Biophys Acta.

[28] Gopal-Srivastava R, Hylemon PB (1988). Purification and characterization of bile salt hydrolase from Clostridium perfringens. J Lipid Res.

[29] Masuda N (1981). Deconjugation of bile salts by bacteroids and clostridium. Microbiol Immunol.

[30] Van Eldere J, Celis P, De Pauw G, Lesaffre E, Eyssen H (1996). Tauroconjugation of cholic acid stimulates 7 alpha-dehydroxylation by fecal bacteria. Appl Environ Microbiol.

[31] Wijaya A, Hermann A, Abriouel H, Specht I, Yousif NM, Holzapfel WH, Franz CM (2004). Cloning of the bile salt hydrolase (bsh) gene from Enterococcus faecium FAIR-E 345 and chromosomal location of bsh genes in food enterococci. J Food Prot.

[32] Jarocki P, Targoński Z (2013). Genetic diversity of bile salt hydrolases among human intestinal bifidobacteria. Curr Microbiol.

[33] Tanaka H, Hashiba H, Kok J, Mierau I (2000). Bile salt hydrolase of Bifidobacterium longum-biochemical and genetic characterization. Appl Environ Microbiol.

[34] De Smet I, Van Hoorde L, VandeWoestyne M, Christiaens H, Verstraete W (1995). Significance of bile salt hydrolytic activities of Lactobacilli. J Appl Microbiol.

[35] Oh HK, Lee JY, Lim SJ, Kim MJ, Kim GB, Kim JH, Hong SK, Kang DK (2008). Molecular cloning and characterization of a bile salt hydrolase from Lactobacillus acidophilus PF01. J Microbiol Biotechnol.

[36] Salvioli G, Salati R, Bondi M, Fratalocchi A, Sala BM, Gibertini A (1982). Bile acid transformation by the intestinal flora and cholesterol saturation in bile effects of Streptococcus faecium administration. Digestion.

[37] Hirano S, Masuda N (1981). Transformation of bile acids by Eubacterium lentum. Appl Environ Microbiol.

[38] Marion S, Desharnais L, Studer N, Dong Y, Notter MD, Poudel S, Menin L, Janowczyk A, Hettich RL, Hapfelmeier S, Bernier-Latmani R (2020). Biogeography of microbial bile acid transformations along the murine gut. J Lipid Res.

[39] Stellwag EJ, Hylemon PB (1976). Purification and characterization of bile salt hydrolase from Bacteroides fragilis subsp. fragilis. Biochim Biophys Acta.

[40] Jones BV, Begley M, Hill C, Gahan CG, Marchesi JR (2008). Functional and comparative metagenomic analysis of bile salt hydrolase activity in the human gut microbiome. Proc Natl Acad Sci USA.

[41] Gerard P (2013). Metabolism of cholesterol and bile acids by the gut microbiota. Pathogens.

[42] Hirano S, Masuda N, Mukai H, Hirakawa K, Imamura T (1979). Transformation of bile acids by Bacteroides fragilis strains isolated from the human intestine (author's transl). Nihon Saikingaku Zasshi.

[43] Long SL, Gahan CGM, Joyce SA (2017). Interactions between gut bacteria and bile in health and disease. Mol Aspects Med.

[44] Ridlon JM, Devendran S, Alves JM, Doden H, Wolf PG, Pereira GV, Ly L, Volland A, Takei H, Nittono H, Murai T, Kurosawa T, Chlipala GE, Green SJ, Hernandez AG, Fields CJ, Wright CL, Kakiyama G, Cann I, Kashyap P, McCracken V, Gaskins HR (2020). The 'in vivo lifestyle' of bile acid 7α-dehydroxylating bacteria: comparative genomics, metatranscriptomic, and bile acid metabolomics analysis of a defined microbial community in gnotobiotic mice x. Gut Microbes.

[45] Ridlon JM, Hylemon PB (2012). Identification and characterization of two bile acid coenzyme A transferases from Clostridium scindens, a bile acid 7α-dehydroxylating intestinal bacterium. J Lipid Res.

[46] Chikai T, Nakao H, Uchida K (1987). Deconjugation of bile acids by human intestinal bacteria implanted in germ-free rats. Lipids.

[47] Narushima S, Itoha K, Miyamoto Y, Park SH, Nagata K, Kuruma K, Uchida K (2006). Deoxycholic acid formation in gnotobiotic mice associated with human intestinal bacteria. Lipids.

[48] Vital M, Rud T, Rath S, Pieper DH, Schlüter D (2019). Diversity of bacteria exhibiting bile acid-inducible 7α-dehydroxylation genes in the human gut, computational and structural. Biotechnol J.

[49] Ramírez-Pérez O, Cruz-Ramón V, Chinchilla-López P, Méndez-Sánchez N (2017). The role of the gut microbiota in bile acid metabolism. Ann Hepatol.

[50] Begley M, Gahan CGM, Hill C (2005). The interaction between bacteria and bile. FEMS Microbiol Rev.

[51] Garcia-Quintanilla M, Prieto AI, Barnes L, Ramos-Morales F, Casadesus J (2006). Bile-induced curing of the virulence plasmid in Salmonella enterica serovar Typhimurium. J Bacteriol.

[52] Merritt ME, Donaldson JR (2009). Effect of bile salts on the DNA and membrane integrity of enteric bacteria. J Med Microbiol.

[53] Prieto AI, Ramos-Morales F, Casadesus J (2004). Bile-induced DNA damage in Salmonella enterica. Genetics.

[54] Schaffler H, Breitruck A (2018). Clostridium difficile—from colonization to infection. Front Microbiol.

[55] Sorg JA, Sonenshein AL (2010). Inhibiting the initiation of Clostridium difficile spore germination using analogs of chenodeoxycholic acid, a bile acid. J Bacteriol.

[56] Tsuei J, Chau T, Mills D, Wan YJ (2014). Bile acid dysregulation, gut dysbiosis, and gastrointestinal cancer. Exp Biol Med.

[57] Slocum MM, Sittig KM, Specian RD, Deitch EA (1992). Absence of intestinal bile promotes bacterial translocation. Am Surg.

[58] van Best N, Rolle-Kampczyk U, Schaap FG, Basic M, Olde Damink SWM, Bleich A, Savelkoul PHM, von Bergen M, Penders J, Hornef MW (2020). Bile acids drive the newborn’s gut microbiota maturation. Nat Commun.

[59] Thomas RM, Jobin C (2015). The microbiome and cancer: is the 'oncobiome' mirage real?. Trends in Cancer.

[60] Miko E, Vida A, Bai P (2016). Translational aspects of the microbiome-to be exploited. Cell Biol Toxicol.

[61] Sári Z, Kovács T, Csonka T, Török M, Sebő É, Toth J, Tóth D, Mikó E, Kiss B, Szeőcs D, Uray K, Karányi Z, Kovács I, Méhes G, Árkosy P, B. P, (2020). Fecal expression of E. coli lysine decarboxylase (LdcC) is downregulated in E-cadherin negative lobular breast carcinoma. Physiol Int.

[62] Sári Z, Mikó E, Kovács T, Boratkó A, Ujlaki G, Jankó L, Kiss B, Uray K, Bai P (2020). Indoxylsulfate, a metabolite of the microbiome has cytostatic effects in breast cancer via activation of AHR and PXR receptors and induction of oxidative stress. Cancers (Basel).

[63] Sári Z, Mikó E, Kovács T, Jankó L, Csonka T, Sebő E, Toth J, Tóth D, Árkosy P, Boratkó A, Ujlaki G, Török M, Kovács I, Szabó J, Kiss B, Méhes G, Goedert JJ, Bai P (2020). Indolepropionic acid, a metabolite of the microbiome, has cytostatic properties in breast cancer by activating AHR and PXR receptors and inducing oxidative stress. Cancers (Basel).

[64] Kovács T, Mikó E, Vida A, Sebő É, Toth J, Csonka T, Boratkó A, Ujlaki G, Lente G, Kovács P, Tóth D, Árkosy P, Kiss B, Méhes G, Goedert JJ, Bai P (2019). Cadaverine, a metabolite of the microbiome, reduces breast cancer aggressiveness through trace amino acid receptors. Sci Rep.

[65] Dawson PA, Lan T, Rao A (2009). Thematic review series: Bile acids Bile acid transporters. Am Soc Biochem Mol Biol.

[66] Claro Da Silva T, Polli JE, Swaan PW (2013). The solute carrier family 10 (SLC10): Beyond bile acid transport.

[67] Keppler D (2017). Progress in the molecular characterization of hepatobiliary transporters. Dig Dis.

[68] Lee W, Glaeser H, Smith LH, Roberts RL, Moeckel GW, Gervasini G, Leake BF, Kim RB (2005). Polymorphisms in human organic anion-transporting polypeptide 1A2 (OATP1A2): implications for altered drug disposition and central nervous system drug entry. J Biol Chem.

[69] Hagenbuch B, Stieger B (2013). The SLCO (former SLC21) superfamily of transporters.

[70] Suga T, Yamaguchi H, Sato T, Maekawa M, Goto J, Mano N (2017). Preference of conjugated bile acids over unconjugated bile acids as substrates for OATP1B1 and OATP1B3. PLoS ONE.

[71] Roth M, Obaidat A, Hagenbuch B (2012). OATPs, OATs and OCTs: The organic anion and cation transporters of the SLCO and SLC22A gene superfamilies.

[72] Makishima M, Okamoto AY, Repa JJ, Tu H, Learned RM, Luk A, Hull MV, Lustig KD, Mangelsdorf DJ, Shan B (1999). Identification of a nuclear receptor for bile acids. Science.

[73] Kawamata Y, Fujii R, Hosoya M, Harada M, Yoshida H, Miwa M, Fukusumi S, Habata Y, Itoh T, Shintani Y, Hinuma S, Fujisawa Y, Fujino M (2003). A G protein-coupled receptor responsive to bile acids. J Biol Chem.

[74] Maruyama T, Miyamoto Y, Nakamura T, Tamai Y, Okada H, Sugiyama E, Nakamura T, Itadani H, Tanaka K (2002). Identification of membrane-type receptor for bile acids (M-BAR). Biochem Biophys Res Commun.

[75] Hang S, Paik D, Yao L, Kim E, Trinath J, Lu J, Ha S, Nelson BN, Kelly SP, Wu L, Zheng Y, Longman RS, Rastinejad F, Devlin AS, Krout MR, Fischbach MA, Littman DR, Huh JR (2019). Bile acid metabolites control TH17 and Treg cell differentiation. Nature.

[76] McIlvride S, Dixon PH, Williamson C (2017). Bile acids and gestation. Mol Aspects Med.

[77] Keitel V, Cupisti K, Ullmer C, Knoefel WT, Kubitz R, Häussinger D (2009). The membrane-bound bile acid receptor TGR5 is localized in the epithelium of human gallbladders. Hepatology.

[78] Poole DP, Godfrey C, Cattaruzza F, Cottrell GS, Kirkland JG, Pelayo JC, Bunnett NW, Corvera CU (2010). Expression and function of the bile acid receptor GpBAR1 (TGR5) in the murine enteric nervous system. Neurogastroenterol Motil.

[79] Keitel V, Donner M, Winandy S, Kubitz R, Häussinger D (2008). Expression and function of the bile acid receptor TGR5 in Kupffer cells. Biochem Biophys Res Commun.

[80] Sato H, Genet C, Strehle A, Thomas C, Lobstein A, Wagner A, Mioskowski C, Auwerx J, Saladin R (2007). Anti-hyperglycemic activity of a TGR5 agonist isolated from Olea europaea. Biochem Biophys Res Commun.

[81] Pellicciari R, Gioiello A, Macchiarulo A, Thomas C, Rosatelli E, Natalini B, Sardella R, Pruzanski M, Roda A, Pastorini E, Schoonjans K, Auwerx J (2009). Discovery of 6alpha-ethyl-23(S)-methylcholic acid (S-EMCA, INT-777) as a potent and selective agonist for the TGR5 receptor, a novel target for diabesity. J Med Chem.

[82] Rizzo G, Passeri D, De Franco F, Ciaccioli G, Donadio L, Rizzo G, Orlandi S, Sadeghpour B, Wang XX, Jiang T, Levi M, Pruzanski M, Adorini L (2010). Functional characterization of the semisynthetic bile acid derivative INT-767, a dual farnesoid X receptor and TGR5 agonist. Mol Pharmacol.

[83] Genet C, Strehle A, Schmidt C, Boudjelal G, Lobstein A, Schoonjans K, Souchet M, Auwerx J, Saladin R, Wagner A (2010). Structure-activity relationship study of betulinic acid, a novel and selective TGR5 agonist, and its synthetic derivatives: potential impact in diabetes. J Med Chem.

[84] Zheng C, Zhou W, Wang T, You P, Zhao Y, Yang Y, Wang X, Luo J, Chen Y, Liu M, Chen H (2015). A novel TGR5 activator WB403 promotes GLP-1 secretion and preserves pancreatic β- Cells in type 2 diabetic mice. PLoS ONE.

[85] Pols TWH, Noriega LG, Nomura M, Auwerx J, Schoonjans K (2011). The bile acid membrane receptor TGR5 as an emerging target in metabolism and inflammation. J Hepatol.

[86] Reich M, Deutschmann K, Sommerfeld A, Klindt C, Kluge S, Kubitz R, Ullmer C, Knoefel WT, Herebian D, Mayatepek E, Häussinger D, Keitel V (2016). TGR5 is essential for bile acid-dependent cholangiocyte proliferation in vivo and in vitro. Gut.

[87] Masyuk AI, Huang BQ, Radtke BN, Gajdos GB, Splinter PL, Masyuk TV, Gradilone SA, LaRusso NF (2013). Ciliary subcellular localization of TGR5 determines the cholangiocyte functional response to bile acid signaling. Am J Physiol Gastrointest Liver Physiol.

[88] Perino A, Pols TWH, Nomura M, Stein S, Pellicciari R, Schoonjans K (2014). TGR5 reduces macrophage migration through mTOR-induced C/EBPβ differential translation. J Clin Investig.

[89] Rajagopal S, Kumar DP, Mahavadi S, Bhattacharya S, Zhou R, Corvera CU, Bunnett NW, Grider JR, Murthy KS (2013). Activation of G protein-coupled bile acid receptor, TGR5, induces smooth muscle relaxation via both Epac- and PKA-mediated inhibition of RhoA/Rho kinase pathway. Am J Physiol Gastrointest Liver Physiol.

[90] Maruyama T, Tanaka K, Suzuki J, Miyoshi H, Harada N, Nakamura T, Miyamoto Y, Kanatani A, Tamai Y (2006). Targeted disruption of G protein-coupled bile acid receptor 1 (Gpbar1/M-Bar) in mice. J Endocrinol.

[91] Guo C, Su J, Li Z, Xiao R, Wen J, Li Y, Zhang M, Zhang X, Yu D, Huang W, Chen WD, Wang YD (2015). The G-protein-coupled bile acid receptor Gpbar1 (TGR5) suppresses gastric cancer cell proliferation and migration through antagonizing STAT3 signaling pathway. Oncotarget.

[92] Wang YD, Chen WD, Yu D, Forman BM, Huang W (2011). The G-Protein-coupled bile acid receptor, Gpbar1 (TGR5), negatively regulates hepatic inflammatory response through antagonizing nuclear factor kappa light-chain enhancer of activated B cells (NF-κB) in mice. Hepatology.

[93] Guo C, Chen WD, Wang YD (2016). TGR5, not only a metabolic regulator. Front Physiol.

[94] Pols TWH, Nomura M, Harach T, Lo Sasso G, Oosterveer MH, Thomas C, Rizzo G, Gioiello A, Adorini L, Pellicciari R, Auwerx J, Schoonjans K (2011). TGR5 activation inhibits atherosclerosis by reducing macrophage inflammation and lipid loading. Cell Metab.

[95] Liu R, Zhao R, Zhou X, Liang X, Campbell DJW, Zhang X, Zhang L, Shi R, Wang G, Pandak WM, Sirica AE, Hylemon PB, Zhou H (2014). Conjugated bile acids promote cholangiocarcinoma cell invasive growth through activation of sphingosine 1-phosphate receptor 2. Hepatology.

[96] Liu R, Li X, Qiang X, Luo L, Hylemon PB, Jiang Z, Zhang L, Zhou H (2015). Taurocholate induces cyclooxygenase-2 expression via the sphingosine 1-phosphate receptor 2 in a human cholangiocarcinoma cell line. J Biol Chem.

[97] Studer E, Zhou X, Zhao R, Wang Y, Takabe K, Nagahashi M, Pandak WM, Dent P, Spiegel S, Shi R, Xu W, Liu X, Bohdan P, Zhang L, Zhou H, Hylemon PB (2012). Conjugated bile acids activate the sphingosine-1-phosphate receptor 2 in primary rodent hepatocytes. Hepatology.

[98] Nagahashi M, Takabe K, Liu R, Peng K, Wang X, Wang Y, Hait NC, Wang X, Allegood JC, Yamada A, Aoyagi T, Liang J, Pandak WM, Spiegel S, Hylemon PB, Zhou H (2015). Conjugated bile acid-activated S1P receptor 2 is a key regulator of sphingosine kinase 2 and hepatic gene expression. Hepatology.

[99] Nagahashi M, Yuza K, Hirose Y, Nakajima M, Ramanathan R, Hait NC, Hylemon PB, Zhou H, Takabe K, Wakai T (2016). The roles of bile acids and sphingosine-1-phosphate signaling in the hepatobiliary diseases. J Lipid Res.

[100] Yang J, Yang L, Tian L, Ji X, Yang L, Li L (2018). Sphingosine 1-phosphate (S1P)/S1P receptor 2/3 axis promotes inflammatory M1 polarization of bone marrow-derived monocyte/macrophage via G(α) i/o /PI3K/JNK pathway. Cell Physiol Biochem.

[101] Karimian G, Buist-Homan M, Schmidt M, Tietge UJF, de Boer JF, Klappe K, Kok JW, Combettes L, Tordjmann T, Faber KN, Moshage H (1832). Sphingosine kinase-1 inhibition protects primary rat hepatocytes against bile salt-induced apoptosis. Biochim Biophys Acta Mol Basis Dis.

[102] Hughes JE, Srinivasan S, Lynch KR, Proia RL, Ferdek P, Hedrick CC (2008). Sphingosine-1-phosphate induces an antiinflammatory phenotype in macrophages. Circ Res.

[103] Grigorova IL, Schwab SR, Phan TG, Pham TH, Okada T, Cyster JG (2009). Cortical sinus probing, S1P1-dependent entry and flow-based capture of egressing T cells. Nat Immunol.

[104] von Rosenvinge EC, Raufman JP (2011). Muscarinic receptor signaling in colon cancer. Cancers.

[105] Cheng K, Chen Y, Zimniak P, Raufman JP, Xiao Y, Frucht H (2002). Functional interaction of lithocholic acid conjugates with M3 muscarinic receptors on a human colon cancer cell line. Biochim Biophys Acta Mol Basis Dis.

[106] Amonyingcharoen S, Suriyo T, Thiantanawat A, Watcharasit P, Satayavivad J (2015). Taurolithocholic acid promotes intrahepatic cholangiocarcinoma cell growth via muscarinic acetylcholine receptor and EGFR/ERK1/2 signaling pathway. Int J Oncol.

[107] Forman BM, Goode E, Chen J, Oro AE, Bradley DJ, Perlmann T, Noonan DJ, Burka LT, McMorris T, Lamph WW, Evans RM, Weinberger C (1995). Identification of a nuclear receptor that is activated by farnesol metabolites. Cell.

[108] Seol W, Choi HS, Moore DD (1996). An orphan nuclear hormone receptor that lacks a DNA binding domain and heterodimerizes with other receptors. Science.

[109] Goodwin B, Jones SA, Price RR, Watson MA, McKee DD, Moore LB, Galardi C, Wilson JG, Lewis MC, Roth ME, Maloney PR, Willson TM, Kliewer SA (2000). A regulatory cascade of the nuclear receptors FXR, SHP-1, and LRH-1 represses bile acid biosynthesis. Mol Cell.

[110] Zhang M, Chiang JYL (2001). Transcriptional regulation of the human sterol 12α-hydroxylase gene (CYP8B1): roles of hepatocyte nuclear factor 4α in mediating bile acid repression. J Biol Chem.

[111] Kong B, Wang L, Chiang JYL, Zhang Y, Klaassen CD, Guo GL (2012). Mechanism of tissue-specific farnesoid X receptor in suppressing the expression of genes in bile-acid synthesis in mice. Hepatology.

[112] Denson LA, Sturm E, Echevarria W, Zimmerman TL, Makishima M, Mangelsdorf DJ, Karpen SJ (2001). The orphan nuclear receptor, shp, mediates bile acid-induced inhibition of the rat bile acid transporter, ntcp. Gastroenterology.

[113] Ananthanarayanan M, Balasubramanian N, Makishima M, Mangelsdorf DJ, Suchy FJ (2001). Human bile salt export pump promoter is transactivated by the Farnesoid X receptor/bile acid receptor. J Biol Chem.

[114] Grobert J, Zaghini I, Fujii H, Jones SA, Kliewer SA, Willson TM, Ono T, Besnard P (1999). Identification of a bile acid-responsive element in the human ileal bile acid-binding protein gene. Involvement of the farnesoid X receptor/9-cis- retinoic acid receptor heterodimer. J Biol Chem.

[115] Gnerre C, Blättler S, Kaufmann MR, Looser R, Meyer UA (2004). Regulation of CYP3A4 by the bile acid receptor FXR: evidence for functional binding sites in the CYP3A4 gene. Pharmacogenetics.

[116] Song CS, Echchgadda I, Baek BS, Ahn SC, Oh T, Roy AK, Chatterjee B (2001). Dehydroepiandrosterone sulfotransferase gene induction by bile acid activated farnesoid X receptor. J Biol Chem.

[117] Barbier O, Torra IP, Sirvent A, Claudel T, Blanquart C, Duran-Sandoval D, Kuipers F, Kosykh V, Fruchart JC, Staels B (2003). FXR induces the UGT2B4 enzyme in hepatocytes: a potential mechanism of negative feedback control of FXR activity. Gastroenterology.

[118] Wang YD, Chen WD, Wang M, Yu D, Forman BM, Huang W (2008). Farnesoid X receptor antagonizes nuclear factor κB in hepatic inflammatory response. Hepatology.

[119] Lamba V, Yasuda K, Lamba JK, Assem M, Davila J, Strom S, Schuetz EG (2004). PXR (NR1I2): splice variants in human tissues, including brain, and identification of neurosteroids and nicotine as PXR activators. Toxicol Appl Pharmacol.

[120] Wang YM, Ong SS, Chai SC, Chen T (2012). Role of CAR and PXR in xenobiotic sensing and metabolism. Expert Opin Drug Metab Toxicol.

[121] He J, Nishida S, Xu M, Makishima M, Xie W (2011). PXR prevents cholesterol gallstone disease by regulating biosynthesis and transport of bile salts. Gastroenterology.

[122] Staudinger JL, Goodwin B, Jones SA, Hawkins-Brown D, MacKenzie KI, LaTour A, Liu Y, Klaassen CD, Brown KK, Reinhard J, Willson TM, Koller BH, Kliewer SA (2001). The nuclear receptor PXR is a lithocholic acid sensor that protects against liver toxicity. Proc Natl Acad Sci USA.

[123] Wistuba W, Gnewuch C, Liebisch G, Schmitz G, Langmann T (2007). Lithocholic acid induction of the FGF19 promoter in intestinal cells is mediated by PXR. World J Gastroenterol.

[124] Xie W, Radominska-Pandya A, Shi Y, Simon CM, Nelson MC, Ong ES, Waxman DJ, Evans RM (2001). An essential role for nuclear receptors SXR/PXR in detoxification of cholestatic bile acids. Proc Natl Acad Sci USA.

[125] Jonker JW, Liddle C, Downes M (2012). FXR and PXR: potential therapeutic targets in cholestasis. J Steroid Biochem Mol Biol.

[126] Li T, Chiang JYL (2005). Mechanism of rifampicin and pregnane X receptor inhibition of human cholesterol 7α-hydroxylase gene transcription. Am J Physiol Gastrointest Liver Physiol.

[127] Wallace K, Cowie DE, Konstantinou DK, Hill SJ, Tjelle TE, Axon A, Koruth M, White SA, Carlsen H, Mann DA, Wright MC (2010). The PXR is a drug target for chronic inflammatory liver disease. J Steroid Biochem Mol Biol.

[128] Kakizaki S, Yamazaki Y, Takizawa D, Negishi M (2008). New insights on the xenobiotic-sensing nuclear receptors in liver diseases–CAR and PXR. Curr Drug Metab.

[129] Cheng J, Shah YM, Gonzalez FJ (2012). Pregnane X receptor as a target for treatment of inflammatory bowel disorders. Trends Pharmacol Sci.

[130] Zhou J, Zhai Y, Mu Y, Gong H, Uppal H, Toma D, Ren S, Evans RM, Xie W (2006). A novel pregnane X receptor-mediated and sterol regulatory element-binding protein-independent lipogenic pathway. J Biol Chem.

[131] Nakamura K, Moore R, Negishi M, Sueyoshi T (2007). Nuclear pregnane X receptor cross-talk with FoxA2 to mediate drug-induced regulation of lipid metabolism in fasting mouse liver. J Biol Chem.

[132] Kodama S, Moore R, Yamamoto Y, Negishi M (2007). Human nuclear pregnane X receptor cross-talk with CREB to repress cAMP activation of the glucose-6-phosphatase gene. Biochemical Journal.

[133] Bhalla S, Ozalp C, Fang S, Xiang L, Kemper JK (2004). Ligand-activated pregnane X receptor interferes with HNF-4 signaling by targeting a common coactivator PGC-1α. Functional implications in hepatic cholesterol and glucose metabolism. J Biol Chem.

[134] Choi HS, Chung M, Tzameli I, Simha D, Lee YK, Seol W, Moore DD (1997). Differential transactivation by two isoforms of the orphan nuclear hormone receptor CAR. J Biol Chem.

[135] Forman BM, Tzameli I, Choi HS, Chen J, Simha D, Seol W, Evans RM, Moore DD (1998). Androstane metabolites bind to and deactivate the nuclear receptor CAR- β. Nature.

[136] Li H, Wang H (2010). Activation of xenobiotic receptors: Driving into the nucleus. Expert Opin Drug Metab Toxicol.

[137] Baes M, Gulick T, Choi HS, Martinoli MG, Simha D, Moore DD (1994). A new orphan member of the nuclear hormone receptor superfamily that interacts with a subset of retinoic acid response elements. Mol Cell Biol.

[138] di Masi A, De Marinis E, Ascenzi P, Marino M (2009). Nuclear receptors CAR and PXR: molecular, functional, and biomedical aspects. Mol Aspects Med.

[139] Wagner M, Halilbasic E, Marschall HU, Zollner G, Fickert P, Langner C, Zatloukal K, Denk H, Trauner M (2005). CAR and PXR agonists stimulate hepatic bile acid and bilirubin detoxification and elimination pathways in mice. Hepatology.

[140] Han S, Chiang JY (2009). Mechanism of vitamin D receptor inhibition of cholesterol 7alpha-hydroxylase gene transcription in human hepatocytes. Drug Metab Dispos.

[141] Li Z, Kar Kruijt J, van der Sluis RJ, Van Berkel TJC, Hoekstra M (2013). Nuclear receptor atlas of female mouse liver parenchymal, endothelial, and Kupffer cells. Physiol Genom.

[142] Norman AW (2006). Minireview: vitamin D receptor: new assignments for an already busy receptor. Endocrinology.

[143] Makishima M, Lu TT, Xie W, Whitfield GK, Domoto H, Evans RM, Haussler MR, Mangelsdorf DJ (2002). Vitamin D receptor as an intestinal bile acid sensor. Science.

[144] Nehring JA, Zierold C, DeLuca HF (2007). Lithocholic acid can carry out in vivo functions of vitamin D. Proc Natl Acad Sci USA.

[145] Cheng J, Fang ZZ, Kim JH, Krausz KW, Tanaka N, Chiang JYL, Gonzalez FJ (2014). Intestinal CYP3A4 protects against lithocholic acid-induced hepatotoxicity in intestine-specific VDR-deficient mice. J Lipid Res.

[146] Chatterjee B, Echchgadda I, Song CS (2005). Vitamin D receptor regulation of the steroid/bile acid sulfotransferase SULT2A1. Methods Enzymol.

[147] McCarthy TC, Li X, Sinal CJ (2005). Vitamin D receptor-dependent regulation of colon multidrug resistance-associated protein 3 gene expression by bile acids. J Biol Chem.

[148] Chen X, Chen F, Liu S, Glaeser H, Dawson PA, Hofmann AF, Kim RB, Shneider BL, Pang KS (2006). Transactivation of rat apical sodium-dependent bile acid transporter and increased bile acid transport by 1alpha,25-dihydroxyvitamin D3 via the vitamin D receptor. Mol Pharmacol.

[149] Huhtakangas JA, Olivera CJ, Bishop JE, Zanello LP, Norman AW (2004). The vitamin D receptor is present in caveolae-enriched plasma membranes and binds 1α,25(OH)2-vitamin D3 in vivo and in vitro. Mol Endocrinol.

[150] Han S, Li T, Ellis E, Strom S, Chiang JY (2010). A novel bile acid-activated vitamin D receptor signaling in human hepatocytes. Mol Endocrinol.

[151] Daldebert E, Biyeyeme MJ, Mve B, Mergey M, Wendum D, Firrincieli D, Coilly A, Fouassier L, Corpechot C, Poupon R, Housset C, Chignard N (2009). Bile salts control the antimicrobial peptide cathelicidin through nuclear receptors in the human biliary epithelium. Gastroenterology.

[152] Nagpal S, Na S, Rathnachalam R (2005). Noncalcemic actions of vitamin D receptor ligands. Endocr Rev.

[153] Janowski BA, Willy PJ, Devi TR, Falck JR, Mangelsdorf DJ (1996). An oxysterol signalling pathway mediated by the nuclear receptor LXR alpha. Nature.

[154] Svensson S, Östberg T, Jacobsson M, Norström C, Stefansson K, Hallén D, Johansson IC, Zachrisson K, Ogg D, Jendeberg L (2003). Crystal structure of the heterodimeric complex of LXRα and RXRβ ligand-binding domains in a fully agonistic conformation. EMBO J.

[155] Venkateswaran A, Laffitte BA, Joseph SB, Mak PA, Wilpitz DC, Edwards PA, Tontonoz P (2000). Control of cellular cholesterol efflux by the nuclear oxysterol receptor LXRα. Proc Natl Acad Sci USA.

[156] Joseph SB, Bradley MN, Castrillo A, Bruhn KW, Mak PA, Pei L, Hogenesch J, O'Connell RM, Cheng G, Saez E, Miller JF, Tontonoz P (2004). LXR-dependent gene expression is important for macrophage survival and the innate immune response. Cell.

[157] De Marino S, Carino A, Masullo D, Finamore C, Marchianò S, Cipriani S, Di Leva FS, Catalanotti B, Novellino E, Limongelli V, Fiorucci S, Zampella A (2017). Hyodeoxycholic acid derivatives as liver X receptor α and G-protein-coupled bile acid receptor agonists. Sci Rep.

[158] Zhang Y, Hagedorn CH, Wang L (1812). Role of nuclear receptor SHP in metabolism and cancer. Biochim Biophys Acta Mol Basis Dis.

[159] Miao J, Xiao Z, Kanamaluru D, Min G, Yau PM, Veenstra TD, Ellis E, Strom S, Suino-Powell K, Xu HE, Kemper JK (2009). Bile acid signaling pathways increase stability of Small Heterodimer Partner (SHP) by inhibiting ubiquitin-proteasomal degradation. Genes Dev.

[160] Miao J, Fang S, Lee J, Comstock C, Knudsen KE, Kemper JK (2009). Functional Specificities of Brm and Brg-1 Swi/Snf ATPases in the Feedback Regulation of Hepatic Bile Acid Biosynthesis. Mol Cell Biol.

[161] Kim KJ, Kim KH, Cho HK, Kim HY, Kim HH, Cheong JH (2010). SHP (small heterodimer partner) suppresses the transcriptional activity and nuclear localization of Hedgehog signalling protein Gli1. Biochemical Journal.

[162] Gotley DC, Morgan AP, Ball D, Owen RW, Cooper MJ (1991). Composition of gastro-oesophageal refluxate. Gut.

[163] Nehra D, Howell P, Williams CP, Pye JK, Beynon J (1999). Toxic bile acids in gastro-oesophageal reflux disease: influence of gastric acidity. Gut.

[164] Hong J, Behar J, Wands J, Resnick M, Wang LJ, DeLellis RA, Lambeth D, Souza RF, Spechler SJ, Cao W (2010). Role of a novel bile acid receptor TGR5 in the development of oesophageal adenocarcinoma. Gut.

[165] Liu R, Li X, Hylemon PB, Zhou H (2018). Conjugated bile acids promote invasive growth of esophageal adenocarcinoma cells and cancer stem cell expansion via sphingosine 1-phosphate receptor 2-mediated yes-associated protein activation. Am J Pathol.

[166] Zhou Z, Xia Y, Bandla S, Zakharov V, Wu S, Peters J, Godfrey TE, Sun J (2014). Vitamin D receptor is highly expressed in precancerous lesions and esophageal adenocarcinoma with significant sex difference. Hum Pathol.

[167] De Gottardi A, Dumonceau JM, Bruttin F, Vonlaufen A, Morard I, Spahr L, Rubbia-Brandt L, Frossard JL, Dinjens WN, Rabinovitch PS, Hadengue A (2006). Expression of the bile acid receptor FXR in Barrett's esophagus and enhancement of apoptosis by guggulsterone in vitro. Mol Cancer.

[168] Guan B, Li H, Yang Z, Hoque A, Xu X (2013). Inhibition of farnesoid X receptor controls esophageal cancer cell growth in vitro and in nude mouse xenografts. Cancer.

[169] Pang C, LaLonde A, Godfrey TE, Que J, Sun J, Wu TT, Zhou Z (2017). Bile salt receptor TGR5 is highly expressed in esophageal adenocarcinoma and precancerous lesions with significantly worse overall survival and gender differences. Clin Exp Gastroenterol.

[170] Capello A, Moons LM, Van de Winkel A, Siersema PD, van Dekken H, Kuipers EJ, Kusters JG (2008). Bile acid-stimulated expression of the farnesoid X receptor enhances the immune response in Barrett esophagus. Am J Gastroenterol.

[171] Bhat AA, Lu H, Soutto M, Capobianco A, Rai P, Zaika A, El-Rifai W (2018). Exposure of Barrett's and esophageal adenocarcinoma cells to bile acids activates EGFR-STAT3 signaling axis via induction of APE1. Oncogene.

[172] Zhang Q, Agoston AT, Pham TH, Zhang W, Zhang X, Huo X, Peng S, Bajpai M, Das K, Odze RD, Spechler SJ, Souza RF (2019). Acidic bile salts induce epithelial to mesenchymal transition via VEGF signaling in non-neoplastic Barrett's cells. Gastroenterology.

[173] Zhou Z, Lu H, Zhu S, Gomaa A, Chen Z, Yan J, Washington K, El-Rifai W, Dang C, Peng D (2019). Activation of EGFR-DNA-PKcs pathway by IGFBP2 protects esophageal adenocarcinoma cells from acidic bile salts-induced DNA damage. J Exp Clin Cancer Res CR.

[174] Correa P, Piazuelo MB (2012). The gastric precancerous cascade. J Dig Dis.

[175] Matsuhisa T, Arakawa T, Watanabe T, Tokutomi T, Sakurai K, Okamura S, Chono S, Kamada T, Sugiyama A, Fujimura Y, Matsuzawa K, Ito M, Yasuda M, Ota H, Haruma K (2013). Relation between bile acid reflux into the stomach and the risk of atrophic gastritis and intestinal metaplasia: a multicenter study of 2283 cases. Dig Endosc.

[176] Yu JH, Zheng JB, Qi J, Yang K, Wu YH, Wang K, Wang CB, Sun XJ (2019). Bile acids promote gastric intestinal metaplasia by upregulating CDX2 and MUC2 expression via the FXR/NF-κB signalling pathway. Int J Oncol.

[177] Xu Y, Watanabe T, Tanigawa T, Machida H, Okazaki H, Yamagami H, Watanabe K, Tominaga K, Fujiwara Y, Oshitani N, Arakawa T (2010). Bile acids induce cdx2 expression through the farnesoid x receptor in gastric epithelial cells. J Clin Biochem Nutr.

[178] Park MJ, Kim KH, Kim HY, Kim K, Cheong J (2008). Bile acid induces expression of COX-2 through the homeodomain transcription factor CDX1 and orphan nuclear receptor SHP in human gastric cancer cells. Carcinogenesis.

[179] Wang X, Sun L, Wang X, Kang H, Ma X, Wang M, Lin S, Liu M, Dai C, Dai Z (2017). Acidified bile acids enhance tumor progression and telomerase activity of gastric cancer in mice dependent on c-Myc expression. Cancer Med.

[180] Wang X, Zhou P, Sun X, Zheng J, Wei G, Zhang L, Wang H, Yao J, Lu S, Jia P (2015). Acidified bile acids increase hTERT expression via c-myc activation in human gastric cancer cells. Oncol Rep.

[181] Ni Z, Min Y, Han C, Yuan T, Lu W, Ashktorab H, Smoot DT, Wu Q, Wu J, Zeng W, Shi Y (2020). TGR5-HNF4alpha axis contributes to bile acid-induced gastric intestinal metaplasia markers expression. Cell Death Discovery.

[182] Yasuda H, Hirata S, Inoue K, Mashima H, Ohnishi H, Yoshiba M (2007). Involvement of membrane-type bile acid receptor M-BAR/TGR5 in bile acid-induced activation of epidermal growth factor receptor and mitogen-activated protein kinases in gastric carcinoma cells. Biochem Biophys Res Commun.

[183] Carino A, Graziosi L, D'Amore C, Cipriani S, Marchiano S, Marino E, Zampella A, Rende M, Mosci P, Distrutti E, Donini A, Fiorucci S (2016). The bile acid receptor GPBAR1 (TGR5) is expressed in human gastric cancers and promotes epithelial-mesenchymal transition in gastric cancer cell lines. Oncotarget.

[184] Cao W, Tian W, Hong J, Li D, Tavares R, Noble L, Moss SF, Resnick MB (2013). Expression of bile acid receptor TGR5 in gastric adenocarcinoma. Am J Physiol Gastrointest Liver Physiol.

[185] Yang HB, Song W, Cheng MD, Fan HF, Gu X, Qiao Y, Lu X, Yu RH, Chen LY (2015). Deoxycholic acid inhibits the growth of BGC-823 gastric carcinoma cells via a p53-mediated pathway. Mol Med Rep.

[186] Song W, Yang HB, Chen P, Wang SM, Zhao LP, Xu WH, Fan HF, Gu X, Chen LY (2013). Apoptosis of human gastric carcinoma SGC-7901 induced by deoxycholic acid via the mitochondrial-dependent pathway. Appl Biochem Biotechnol.

[187] Guo C, Qi H, Yu Y, Zhang Q, Su J, Yu D, Huang W, Chen WD, Wang YD (2015). The G-protein-coupled bile acid receptor Gpbar1 (TGR5) inhibits gastric inflammation through antagonizing NF-κB signaling pathway. Front Pharmacol.

[188] Fukase K, Ohtsuka H, Onogawa T, Oshio H, Ii T, Mutoh M, Katayose Y, Rikiyama T, Oikawa M, Motoi F, Egawa S, Abe T, Unno M (2008). Bile acids repress E-cadherin through the induction of Snail and increase cancer invasiveness in human hepatobiliary carcinoma. Cancer Sci.

[189] Kainuma M, Takada I, Makishima M, Sano K (2018). Farnesoid X receptor activation enhances transforming growth factor β-induced epithelial-mesenchymal transition in hepatocellular carcinoma cells. Int J Mol Sci.

[190] Hu Y, Chau T, Liu HX, Liao D, Keane R, Nie Y, Yang H, Wan YJY (2015). Bile acids regulate nuclear receptor (Nur77) expression and intracellular location to control proliferation and apoptosis. Mol Cancer Res.

[191] Jang ES, Yoon JH, Lee SH, Lee SM, Lee JH, Yu SJ, Kim YJ, Lee HS, Kim CY (2014). Sodium taurocholate cotransporting polypeptide mediates dual actions of deoxycholic acid in human hepatocellular carcinoma cells: Enhanced apoptosis versus growth stimulation. J Cancer Res Clin Oncol.

[192] Nguyen PT, Kanno K, Pham QT, Kikuchi Y, Kakimoto M, Kobayashi T, Otani Y, Kishikawa N, Miyauchi M, Arihiro K, Ito M, Tazuma S (2020). Senescent hepatic stellate cells caused by deoxycholic acid modulates malignant behavior of hepatocellular carcinoma. J Cancer Res Clin Oncol.

[193] Xu Z, Huang G, Gong W, Zhou P, Zhao Y, Zhang Y, Zeng Y, Gao M, Pan Z, He F (2012). FXR ligands protect against hepatocellular inflammation via SOCS3 induction. Cell Signal.

[194] Langhi C, Pedraz-Cuesta E, Donate Y, Marrero PF, Haro D, Rodríguez JC (2013). Regulation of N-Myc downstream regulated gene 2 by bile acids. Biochem Biophys Res Commun.

[195] Lee S, Cho YY, Cho EJ, Yu SJ, Lee JH, Yoon JH, Kim YJ (2018). Synergistic effect of ursodeoxycholic acid on the antitumor activity of sorafenib in hepatocellular carcinoma cells via modulation of STAT3 and ERK, Internaltion. J Mol Med.

[196] Liu H, Qin CY, Han GQ, Xu HW, Meng M, Yang Z (2007). Mechanism of apoptotic effects induced selectively by ursodeoxycholic acid on human hepatoma cell lines. World J Gastroenterol.

[197] Zhu L, Shan LJ, Liu YJ, Chen D, Xiao XG, Li Y (2014). Ursodeoxycholic acid induces apoptosis of hepatocellular carcinoma cells in vitro. J Dig Dis.

[198] Chung GE, Yoon JH, Lee JH, Kim HY, Myung SJ, Yu SJ, Lee SH, Lee SM, Kim YJ, Lee HS (2011). Ursodeoxycholic acid-induced inhibition of DLC1 protein degradation leads to suppression of hepatocellular carcinoma cell growth. Oncol Rep.

[199] Lim SC, Choi JE, Kang HS, Si H (2010). Ursodeoxycholic acid switches oxaliplatin-induced necrosis to apoptosis by inhibiting reactive oxygen species production and activating p53-caspase 8 pathway in HepG2 hepatocellular carcinoma. Int J Cancer.

[200] Yang CS, Yuk JM, Kim JJ, Hwang JH, Lee CH, Kim JM, Oh GT, Choi HS, Jo EK (2013). Small heterodimer partner-targeting therapy inhibits systemic inflammatory responses through mitochondrial uncoupling protein 2. PLoS ONE.

[201] Zollner G, Wagner M, Fickert P, Silbert D, Fuchsbichler A, Zatloukal K, Denk H, Trauner M (2005). Hepatobiliary transporter expression in human hepatocellular carcinoma. Liver Int.

[202] Halilbasic E, Claudel T, Trauner M (2013). Bile acid transporters and regulatory nuclear receptors in the liver and beyond. J Hepatol.

[203] Arab JP, Karpen SJ, Dawson PA, Arrese M, Trauner M (2017). Bile acids and nonalcoholic fatty liver disease: Molecular insights and therapeutic perspectives. Hepatology.

[204] Zhang W, Zhou L, Yin P, Wang J, Lu X, Wang X, Chen J, Lin X, Xu G (2015). A weighted relative difference accumulation algorithm for dynamic metabolomics data: long-term elevated bile acids are risk factors for hepatocellular carcinoma. Sci Rep.

[205] Knisely AS, Strautnieks SS, Meier Y, Stieger B, Byrne JA, Portmann BC, Bull LN, Pawlikowska L, Bilezikçi B, Ozçay F, László A, Tiszlavicz L, Moore L, Raftos J, Arnell H, Fischler B, Németh A, Papadogiannakis N, Cielecka-Kuszyk J, Jankowska I, Pawłowska J, Melín-Aldana H, Emerick KM, Whitington PF, Mieli-Vergani G, Thompson RJ (2006). Hepatocellular carcinoma in ten children under five years of age with bile salt export pump deficiency. Hepatology.

[206] Yang F, Huang X, Yi T, Yen Y, Moore DD, Huang W (2007). Spontaneous development of liver tumors in the absence of the bile acid receptor farnesoid X receptor. Can Res.

[207] Degirolamo C, Modica S, Vacca M, Di Tullio G, Morgano A, D'Orazio A, Kannisto K, Parini P, Moschetta A (2015). Prevention of spontaneous hepatocarcinogenesis in farnesoid X receptor-null mice by intestinal-specific farnesoid X receptor reactivation. Hepatology.

[208] Wolfe A, Thomas A, Edwards G, Jaseja R, Guo GL, Apte U (2011). Increased activation of the Wnt/β-catenin pathway in spontaneous hepatocellular carcinoma observed in farnesoid X receptor knockout mice. J Pharmacol Exp Ther.

[209] Nomoto M, Miyata M, Yin S, Kurata Y, Shimada M, Yoshinari K, Gonzalez FJ, Suzuki K, Shibasaki S, Kurosawa T, Yamazoe Y (2009). Bile acid-induced elevated oxidative stress in the absence of farnesoid X receptor. Biol Pharm Bull.

[210] Smolková K, Mikó E, Kovács T, Leguina-Ruzzi A, Sipos A, Bai P (2020). NRF2 in regulating cancer metabolism. Antioxid Redox Signal.

[211] Kong B, Zhu Y, Li G, Williams JA, Buckley K, Tawfik O, Luyendyk JP, Guo GL (2016). Mice with hepatocyte-specific FXR deficiency are resistant to spontaneous but susceptible to cholic acid-induced hepatocarcinogenesis. Am J Physiol Gastrointestinal and Liver Physiology.

[212] Jia W, Xie G, Jia W (2018). Bile acid-microbiota crosstalk in gastrointestinal inflammation and carcinogenesis. Nat Rev Gastroenterol Hepatol.

[213] Bernstein H, Bernstein C, Payne CM, Dvorakova K, Garewal H (2005). Bile acids as carcinogens in human gastrointestinal cancers. Mutat Res.

[214] Gadaleta RM, Oldenburg B, Willemsen EC, Spit M, Murzilli S, Salvatore L, Klomp LW, Siersema PD, van Erpecum KJ, van Mil SW (1812). Activation of bile salt nuclear receptor FXR is repressed by pro-inflammatory cytokines activating NF-κB signaling in the intestine. Biochem Biophys Acta.

[215] Zhang Y, Xu P, Park K, Choi Y, Moore DD, Wang L (2008). Orphan receptor small heterodimer partner suppresses tumorigenesis by modulating cyclin D1 expression and cellular proliferation. Hepatology.

[216] Zhang Y, Soto J, Park K, Viswanath G, Kuwada S, Abel ED, Wang L (2010). Nuclear receptor SHP, a death receptor that targets mitochondria, induces apoptosis and inhibits tumor growth. Mol Cell Biol.

[217] He N, Park K, Zhang Y, Huang J, Lu S, Wang L (2008). Epigenetic inhibition of nuclear receptor small heterodimer partner is associated with and regulates hepatocellular carcinoma growth. Gastroenterology.

[218] Yang CS, Kim JJ, Kim TS, Lee PY, Kim SY, Lee HM, Shin DM, Nguyen LT, Lee MS, Jin HS, Kim KK, Lee CH, Kim MH, Park SG, Kim JM, Choi HS, Jo EK (2015). Small heterodimer partner interacts with NLRP3 and negatively regulates activation of the NLRP3 inflammasome. Nat Communun.

[219] Gandhi D, Ojili V, Nepal P, Nagar A, Hernandez-Delima FJ, Bajaj D, Choudhary G, Gupta N, Sharma P (2020). A pictorial review of gall stones and its associated complications. Clin Imaging.

[220] Feng HY, Chen YC (2016). Role of bile acids in carcinogenesis of pancreatic cancer: An old topic with new perspective. World J Gastroenterol.

[221] Fu H, Li Y, Bai G, Yin R, Yin C, Shi W, Zhang L, Li R, Zhao R (2019). Persistent cholestasis resulting from duodenal papillary carcinoma in an adolescent male: A case report. Medicine.

[222] Thomas RM, Jobin C (2020). Microbiota in pancreatic health and disease: the next frontier in microbiome research, Nature Reviews. Gastroenterol Hepatol.

[223] Rees DO, Crick PJ, Jenkins GJ, Wang Y, Griffiths WJ, Brown TH, Al-Sarireh B (2017). Comparison of the composition of bile acids in bile of patients with adenocarcinoma of the pancreas and benign disease. J Steroid Biochem Mol Biol.

[224] Adachi T, Tajima Y, Kuroki T, Mishima T, Kitasato A, Fukuda K, Tsutsumi R, Kanematsu T (2006). Bile-reflux into the pancreatic ducts is associated with the development of intraductal papillary carcinoma in hamsters. J Surg Res.

[225] Tucker ON, Dannenberg AJ, Yang EY, Fahey Iii TJ (2004). Bile acids induce cyclooxygenase-2 expression in human pancreatic cancer cell lines. Carcinogenesis.

[226] Nagathihalli NS, Beesetty Y, Lee W, Washington MK, Chen X, Lockhart AC, Merchant NB (2014). Novel mechanistic insights into ectodomain shedding of EGFR Ligands Amphiregulin and TGF-α: impact on gastrointestinal cancers driven by secondary bile acids. Can Res.

[227] Kim YJ, Jeong SH, Kim EK, Kim EJ, Cho JH (2017). Ursodeoxycholic acid suppresses epithelial-mesenchymal transition and cancer stem cell formation by reducing the levels of peroxiredoxin II and reactive oxygen species in pancreatic cancer cells. Oncol Rep.

[228] Reddy BS, Wynder EL (1977). Metabolic epidemiology of colon cancer Fecal bile acids and neutral sterols in colon cancer patients and patients with adenomatous polyps. Cancer.

[229] Murakami Y, Tanabe S, Suzuki T (2016). High-fat diet-induced intestinal hyperpermeability is associated with increased bile acids in the large intestine of mice. J Food Sci.

[230] Lagergren J, Ye W, Ekbom A (2001). Intestinal cancer after cholecystectomy: is bile involved in carcinogenesis?. Gastroenterology.

[231] Payne CM, Bernstein C, Dvorak K, Bernstein H (2008). Hydrophobic bile acids, genomic instability, Darwinian selection, and colon carcinogenesis. Clin Exp Gastroenterol.

[232] Degirolamo C, Modica S, Palasciano G, Moschetta A (2011). Bile acids and colon cancer: solving the puzzle with nuclear receptors. Trends Mol Med.

[233] Turner DJ, Alaish SM, Zou T, Rao JN, Wang JY, Strauch ED (2007). Bile salts induce resistance to apoptosis through NF-kappaB-mediated XIAP expression. Ann Surg.

[234] Ridlon JM, Wolf PG, Gaskins HR (2016). Taurocholic acid metabolism by gut microbes and colon cancer. Gut Microbes.

[235] Huang XP, Fan XT, Desjeux JF, Castagna M (1992). Bile acids, non-phorbol-ester-type tumor promoters, stimulate the phosphorylation of protein kinase C substrates in human platelets and colon cell line HT29. Int J Cancer.

[236] Moschetta A, Portincasa P, van Erpecum KJ, Debellis L, Vanberge-Henegouwen GP, Palasciano G (2003). Sphingomyelin protects against apoptosis and hyperproliferation induced by deoxycholate: potential implications for colon cancer. Dig Dis Sci.

[237] Zimber A, Gespach C (2008). Bile acids and derivatives, their nuclear receptors FXR, PXR and ligands: role in health and disease and their therapeutic potential. Anticancer Agents Med Chem.

[238] Farhana L, Nangia-Makker P, Arbit E, Shango K, Sarkar S, Mahmud H, Hadden T, Yu Y, Majumdar AP (2016). Bile acid: a potential inducer of colon cancer stem cells. Stem Cell Res Ther.

[239] Hori T, Matsumoto K, Sakaitani Y, Sato M, Morotomi M (1998). Effect of dietary deoxycholic acid and cholesterol on fecal steroid concentration and its impact on the colonic crypt cell proliferation in azoxymethane-treated rats. Cancer Lett.

[240] Di Ciaula A, Garruti G, Lunardi Baccetto R, Molina-Molina E, Bonfrate L, Wang DQ, Portincasa P (2017). Bile acid physiology. Ann Hepatol.

[241] Di Ciaula A, Wang DQ, Molina-Molina E, Lunardi Baccetto R, Calamita G, Palmieri VO, Portincasa P (2017). Bile acids and cancer: direct and environmental-dependent effects. Ann Hepatol.

[242] Zeng H, Claycombe KJ, Reindl KM (2015). Butyrate and deoxycholic acid play common and distinct roles in HCT116 human colon cell proliferation. J Nutr Biochem.

[243] Hess LM, Krutzsch MF, Guillen J, Chow HH, Einspahr J, Batta AK, Salen G, Reid ME, Earnest DL, Alberts DS (2004). Results of a phase I multiple-dose clinical study of ursodeoxycholic acid. Cancer Epidemiol Biomark Prev.

[244] Kim EK, Cho JH, Kim EJ, Kim YJ (2017). Ursodeoxycholic acid inhibits the proliferation of colon cancer cells by regulating oxidative stress and cancer stem-like cell growth. PLoS ONE.

[245] Ochsenkuhn T, Marsteller I, Hay U, Diebold J, Paumgartner G, Goke B, Sackmann M (2003). Does ursodeoxycholic acid change the proliferation of the colorectal mucosa? A randomized, placebo-controlled study. Digestion.

[246] Im E, Martinez JD (2004). Diet induced changes in the colonic environment and colorectal cancer ursodeoxycholic acid (UDCA) can inhibit deoxycholic acid (DCA)-induced apoptosis via modulation of EGFR/ Raf-1/ERK signaling in human colon cancer cells. J Nutr.

[247] Rigas B, Tsioulias GJ, Allan C, Wali RK, Brasitus TA (1994). The effect of bile acids and piroxicam on MHC antigen expression in rat colonocytes during colon cancer development. Immunology.

[248] Alberts DS, Martinez ME, Hess LM, Einspahr JG, Green SB, Bhattacharyya AK, Guillen J, Krutzsch M, Batta AK, Salen G, Fales L, Koonce K, Parish D, Clouser M, Roe D, Lance P (2005). Gastroenterologist, Phase III trial of ursodeoxycholic acid to prevent colorectal adenoma recurrence. J Natl Cancer Inst.

[249] Tung BY, Emond MJ, Haggitt RC, Bronner MP, Kimmey MB, Kowdley KV, Brentnall TA (2001). Ursodiol use is associated with lower prevalence of colonic neoplasia in patients with ulcerative colitis and primary sclerosing cholangitis. Ann Intern Med.

[250] Serfaty L, De Leusse A, Rosmorduc O, Desaint B, Flejou JF, Chazouilleres O, Poupon RE, Poupon R (2003). Ursodeoxycholic acid therapy and the risk of colorectal adenoma in patients with primary biliary cirrhosis: an observational study. Hepatology.

[251] Garrett WS (2015). Cancer and the microbiota. Science.

[252] Tjalsma H, Boleij A, Marchesi JR, Dutilh BE (2012). A bacterial driver-passenger model for colorectal cancer: beyond the usual suspects. Nat Rev Microbiol.

[253] Raskov H, Burcharth J, Pommergaard HC (2017). Linking gut microbiota to colorectal cancer. J Cancer.

[254] Ward JBJ, Lajczak NK, Kelly OB, O'Dwyer AM, Giddam AK, Ni Gabhann J, Franco P, Tambuwala MM, Jefferies CA, Keely S, Roda A, Keely SJ (2017). Ursodeoxycholic acid and lithocholic acid exert anti-inflammatory actions in the colon. Am J Physiol Gastrointest Liver Physiol.

[255] Islam KB, Fukiya S, Hagio M, Fujii N, Ishizuka S, Ooka T, Ogura Y, Hayashi T, Yokota A (2011). Bile acid is a host factor that regulates the composition of the cecal microbiota in rats. Gastroenterology.

[256] Devkota S, Wang Y, Musch MW, Leone V, Fehlner-Peach H, Nadimpalli A, Antonopoulos DA, Jabri B, Chang EB (2012). Dietary-fat-induced taurocholic acid promotes pathobiont expansion and colitis in Il10-/- mice. Nature.

[257] Javitt NB, Budai K, Miller DG, Cahan AC, Raju U, Levitz M (1994). Breast-gut connection: origin of chenodeoxycholic acid in breast cyst fluid. Lancet.

[258] Tang W, Putluri V, Ambati CR, Dorsey TH, Putluri N, Ambs S (2019). Liver- and microbiome-derived bile acids accumulate in human breast tumors and inhibit growth and improve patient survival. Clin Cancer Res.

[259] Murray WR, Blackwood A, Calman KC, MacKay C (1980). Faecal bile acids and clostridia in patients with breast cancer. Br J Cancer.

[260] Luo C, Zhang X, He Y, Chen H, Liu M, Wang H, Tang L, Tu G, Ding M (2021). A pseudo-targeted metabolomics study based on serum bile acids profiling for the differential diagnosis of benign and malignant breast lesions. Steroids.

[261] Raju U, Levitz M, Javitt NB (1990). Bile acids in human breast cyst fluid: the identification of lithocholic acid. J Clin Endocrinol Metab.

[262] Costarelli V, Sanders TA (2002). Plasma bile acids and risk of breast cancer. IARC Sci Publ.

[263] Costarelli V, Sanders TA (2002). Plasma deoxycholic acid concentration is elevated in postmenopausal women with newly diagnosed breast cancer. Eur J Clin Nutr.

[264] Tang X, Lin CC, Spasojevic I, Iversen ES, Chi JT, Marks JR (2014). A joint analysis of metabolomics and genetics of breast cancer. Breast Cancer Res.

[265] Swales KE, Korbonits M, Carpenter R, Walsh DT, Warner TD, Bishop-Bailey D (2006). The farnesoid X receptor is expressed in breast cancer and regulates apoptosis and aromatase expression. Can Res.

[266] Kovács T, Mikó E, Ujlaki G, Yousef H, Csontos V, Uray K, Bai P (2022). The involvement of oncobiosis and bacterial metabolite signaling in metastasis formation in breast cancer. Cancer Metastasis Rev.

[267] Baker PR, Wilton JC, Jones CE, Stenzel DJ, Watson N, Smith GJ (1992). Bile acids influence the growth, oestrogen receptor and oestrogen-regulated proteins of MCF-7 human breast cancer cells. Br J Cancer.

[268] Giordano C, Catalano S, Panza S, Vizza D, Barone I, Bonofiglio D, Gelsomino L, Rizza P, Fuqua SAW, Andò S (2011). Farnesoid X receptor inhibits tamoxifen-resistant MCF-7 breast cancer cell growth through downregulation of HER2 expression. Oncogene.

[269] Journe F, Durbecq V, Chaboteaux C, Rouas G, Laurent G, Nonclercq D, Sotiriou C, Body JJ, Larsimont D (2009). Association between farnesoid X receptor expression and cell proliferation in estrogen receptor-positive luminal-like breast cancer from postmenopausal patients. Breast Cancer Res Treat.

[270] Liu N, Zhao J, Wang J, Teng H, Fu Y, Yuan H (2016). Farnesoid X receptor ligand CDCA suppresses human prostate cancer cells growth by inhibiting lipid metabolism via targeting sterol response element binding protein, American Journal of. Transl Res.

[271] Liu J, Tong SJ, Wang X, Qu LX (2014). Farnesoid X receptor inhibits LNcaP cell proliferation via the upregulation of PTEN. Exp Ther Med.

[272] Kaeding J, Bouchaert E, Bélanger J, Caron P, Chouinard S, Verreault M, Larouche O, Pelletier G, Staels B, Bélanger A, Barbier O (2008). Activators of the farnesoid X receptor negatively regulate androgen glucuronidation in human prostate cancer LNCAP cells. Biochem J.

[273] Goldberg AA, Titorenko VI, Beach A, Sanderson JT (2013). Bile acids induce apoptosis selectively in androgen-dependent and -independent prostate cancer cells. PeerJ.

[274] Lee WS, Jung JH, Panchanathan R, Yun JW, Kim DH, Kim HJ, Kim GS, Ryu CH, Shin SC, Hong SC, Choi YH, Jung J-M (2017). Ursodeoxycholic acid induces death receptor-mediated apoptosis in prostate cancer cells. j Cancer Prev.

[275] Ke C, Hou Y, Zhang H, Fan L, Ge T, Guo B, Zhang F, Yang K, Wang J, Lou G, Li K (2015). Large-scale profiling of metabolic dysregulation in ovarian cancer. Int J Cancer.

[276] Fan L, Yin M, Ke C, Ge T, Zhang G, Zhang W, Zhou X, Lou G, Li K (2016). Use of plasma metabolomics to identify diagnostic biomarkers for early stage epithelial ovarian cancer. J Cancer.

[277] Zhou M, Guan W, Walker LD, Mezencev R, Benigno BB, Gray A, Fernández FM, McDonald JF (2010). Rapid mass spectrometric metabolic profiling of blood sera detects ovarian cancer with high accuracy. Cancer Epidemiol Biomark Prev.

[278] Guan W, Zhou M, Hampton CY, Benigno BB, Walker LD, Gray A, McDonald JF, Fernández FM (2009). Ovarian cancer detection from metabolomic liquid chromatography/mass spectrometry data by support vector machines. BMC Bioinformatics.

[279] Horowitz NS, Hua J, Powell MA, Gibb RK, Mutch DG, Herzog TJ (2007). Novel cytotoxic agents from an unexpected source: bile acids and ovarian tumor apoptosis. Gynecol Oncol.

[280] Schuldes H, Dolderer JH, Zimmer G, Knobloch J, Bickeböller R, Jonas D, Woodcock BG (2001). Reversal of multidrug resistance and increase in plasma membrane fluidity in CHO cells with R-verapamil and bile salts. Eur J Cancer.

[281] Jin Q, Noel O, Nguyen M, Sam L, Gerhard GS (2018). Bile acids upregulate BRCA1 and downregulate estrogen receptor 1 gene expression in ovarian cancer cells. Eur J Cancer Prev.

[282] Pascual MJ, Macias RI, Garcia-Del-Pozo J, Serrano MA, Marin JJ (2001). Enhanced efficiency of the placental barrier to cisplatin through binding to glycocholic acid. Anticancer Res.

[283] Rough JJ, Monroy MA, Yerrum S, Daly JM (2010). Anti-proliferative effect of LXR agonist T0901317 in ovarian carcinoma cells. J Ovar Res.

[284] Scoles DR, Xu X, Wang H, Tran H, Taylor-Harding B, Li A, Karlan BY (2010). Liver X receptor agonist inhibits proliferation of ovarian carcinoma cells stimulated by oxidized low density lipoprotein. Gynecol Oncol.

[285] Curtarello M, Tognon M, Venturoli C, Silic-Benussi M, Grassi A, Verza M, Minuzzo S, Pinazza M, Brillo V, Tosi G, Ferrazza R, Guella G, Iorio E, Godfroid A, Sounni NE, Amadori A, Indraccolo S (2019). Rewiring of lipid metabolism and storage in ovarian cancer cells after anti-VEGF therapy. Cells.

[286] Masuyama H, Nakamura K, Nobumoto E, Hiramatsu Y (2016). Inhibition of pregnane X receptor pathway contributes to the cell growth inhibition and apoptosis of anticancer agents in ovarian cancer cells. Int J Oncol.

[287] Bandera Merchan B, Morcillo S, Martin-Nuñez G, Tinahones FJ, Macías-González M (2017). The role of vitamin D and VDR in carcinogenesis: Through epidemiology and basic sciences. J Steroid Biochem Mol Biol.

[288] Hou YF, Gao SH, Wang P, Zhang HM, Liu LZ, Ye MX, Zhou GM, Zhang ZL, Li BY (2016). 1α,25(OH)_2_D_3_ suppresses the migration of ovarian cancer SKOV-3 cells through the inhibition of epithelial–mesenchymal transition. Int J Mol Sci.

[289] Ji M, Liu L, Hou Y, Li B (2019). 1α,25-Dihydroxyvitamin D3 restrains stem cell-like properties of ovarian cancer cells by enhancing vitamin D receptor and suppressing CD44. Oncol Rep.

[290] Li J, Li B, Jiang Q, Zhang Y, Liu A, Wang H, Zhang J, Qin Q, Hong Z, Li BA (2018). Do genetic polymorphisms of the vitamin D receptor contribute to breast/ovarian cancer? A systematic review and network meta-analysis. Gene.

[291] Lungchukiet P, Sun Y, Kasiappan R, Quarni W, Nicosia SV, Zhang X, Bai W (2015). Suppression of epithelial ovarian cancer invasion into the omentum by 1α,25-dihydroxyvitamin D3 and its receptor. J Steroid Biochem Mol Biol.

[292] Silvagno F, Poma CB, Realmuto C, Ravarino N, Ramella A, Santoro N, D'Amelio P, Fuso L, Pescarmona G, Zola P (2010). Analysis of vitamin D receptor expression and clinical correlations in patients with ovarian cancer. Gynecol Oncol.

[293] Tamez S, Norizoe C, Ochiai K, Takahashi D, Shimojima A, Tsutsumi Y, Yanaihara N, Tanaka T, Okamoto A, Urashima M (2009). Vitamin D receptor polymorphisms and prognosis of patients with epithelial ovarian cancer. Br J Cancer.

[294] Cordes T, Hoellen F, Dittmer C, Salehin D, Kümmel S, Friedrich M, Köster F, Becker S, Diedrich K, Thill M (2012). Correlation of prostaglandin metabolizing enzymes and serum PGE2 levels with vitamin D receptor and serum 25(OH)2D3 levels in breast and ovarian cancer. Anticancer Res.

[295] Moore RG, Lange TS, Robinson K, Kim KK, Uzun A, Horan TC, Kawar N, Yano N, Chu SR, Mao Q, Brard L, DePaepe ME, Padbury JF, Arnold LA, Brodsky A, Shen TL, Singh RK (2012). Efficacy of a non-hypercalcemic vitamin-D2 derived anti-cancer agent (MT19c) and inhibition of fatty acid synthesis in an ovarian cancer xenograft model. PLoS ONE.

[296] Czogalla B, Deuster E, Liao Y, Mayr D, Schmoeckel E, Sattler C, Kolben T, Hester A, Furst S, Burges A, Mahner S, Jeschke U, Trillsch F (2020). Cytoplasmic VDR expression as an independent risk factor for ovarian cancer. Histochem Cell Biol.

[297] Chen Y, Tang Y, Guo C, Wang J, Boral D, Nie D (2012). Nuclear receptors in the multidrug resistance through the regulation of drug-metabolizing enzymes and drug transporters. Biochem Pharmacol.

[298] Wang Y, Masuyama H, Nobumoto E, Zhang G, Hiramatsu Y (2014). The inhibition of constitutive androstane receptor-mediated pathway enhances the effects of anticancer agents in ovarian cancer cells. Biochem Pharmacol.

[299] Gupta D, Venkatesh M, Wang H, Kim S, Sinz M, Goldberg GL, Whitney K, Longley C, Mani S (2008). Expanding the roles for pregnane X receptor in cancer: proliferation and drug resistance in ovarian cancer. Clin Cancer Res.

[300] Szanto M, Gupte R, Kraus WL, Pacher P, Bai P (2021). PARPs in lipid metabolism and related diseases. Progr Lipid Res.

[301] Phelan JP, Reen FJ, Caparros-Martin JA, O'Connor R, O'Gara F (2017). Rethinking the bile acid/gut microbiome axis in cancer. Oncotarget.

[302] Amaral JD, Viana RJS, Ramalho RM, Steer CJ, Rodrigues CMP (2009). Bile acids: Regulation of apoptosis by ursodeoxycholic acid. J Lipid Res.

[303] Trottier J, Białek A, Caron P, Straka RJ, Milkiewicz P, Barbier O (2011). Profiling circulating and urinary bile acids in patients with biliary obstruction before and after biliary stenting. PLoS ONE.

[304] García-Cañaveras JC, Donato MT, Castell JV, Lahoz A (2012). Targeted profiling of circulating and hepatic bile acids in human, mouse, and rat using a UPLC-MRM-MS-validated method. J Lipid Res.

[305] Ma Z, Wang X, Yin P, Wu R, Zhou L, Xu G, Niu J (2019). Serum metabolome and targeted bile acid profiling reveals potential novel biomarkers for drug-induced liver injury. Medicine (Baltimore).

[306] Sun Z, Huang C, Shi Y, Wang R, Fan J, Yu Y, Zhang Z, Zhu K, Li M, Ni Q, Chen Z, Zheng M, Yang Z (2021). Distinct bile acid profiles in patients with chronic hepatitis b virus infection reveal metabolic interplay between host, virus and gut microbiome. Front Med.

[307] James SC, Fraser K, Young W, Heenan PE, Gearry RB, Keenan JI, Talley NJ, Joyce SA, McNabb WC, Roy NC (2021). Concentrations of fecal bile acids in participants with functional gut disorders and healthy controls. Metabolites.

[308] Wei W, Wang HF, Zhang Y, Zhang YL, Niu BY, Yao SK (2020). Altered metabolism of bile acids correlates with clinical parameters and the gut microbiota in patients with diarrhea-predominant irritable bowel syndrome. World J Gastroenterol.

[309] Sergeev I, Keren N, Naftali T, Konikoff FM (2020). Cholecystectomy and biliary sphincterotomy increase fecal bile loss and improve lipid profile in dyslipidemia. Dig Dis Sci.

[310] Zhao A, Wang S, Chen W, Zheng X, Huang F, Han X, Ge K, Rajani C, Huang Y, Yu H, Zhu J, Jia W (2020). Increased levels of conjugated bile acids are associated with human bile reflux gastritis. Sci Rep.

[311] Jäntti SE, Kivilompolo M, Ohrnberg L, Pietiläinen KH, Nygren H, Orešič M, Hyötyläinen T (2014). Quantitative profiling of bile acids in blood, adipose tissue, intestine, and gall bladder samples using ultra high performance liquid chromatography-tandem mass spectrometry. Anal Bioanal Chem.

[312] Setchell KD, Rodrigues CM, Clerici C, Solinas A, Morelli A, Gartung C, Boyer J (1997). Bile acid concentrations in human and rat liver tissue and in hepatocyte nuclei. Gastroenterology.

[313] Honda A, Yoshida T, Tanaka N, Matsuzaki Y, He B, Shoda J, Osuga T (1995). Increased bile acid concentration in liver tissue with cholesterol gallstone disease. J Gastroenterol Hepatol.

[314] Yao Z, Zhang X, Zhao F, Wang S, Chen A, Huang B, Wang J, Li X (2020). Ursodeoxycholic acid inhibits glioblastoma progression via endoplasmic reticulum stress related apoptosis and synergizes with the proteasome inhibitor bortezomib. ACS Chem Neurosci.

[315] Fonseca I, Gordino G, Moreira S, Nunes MJ, Azevedo C, Gama MJ, Rodrigues E, Rodrigues CMP, Castro-Caldas M (2017). Tauroursodeoxycholic acid protects against mitochondrial dysfunction and cell death via mitophagy in human neuroblastoma cells. Mol Neurobiol.

[316] Yu H, Fu QR, Huang ZJ, Lin JY, Chen QX, Wang Q, Shen DY (2019). Apoptosis induced by ursodeoxycholic acid in human melanoma cells through the mitochondrial pathway. Oncol Rep.

[317] Liu H, Xu HW, Zhang YZ, Huang Y, Han GQ, Liang TJ, Wei LL, Qin CY, Qin CK (2015). Ursodeoxycholic acid induces apoptosis in hepatocellular carcinoma xenografts in mice. World J Gastroenterol.

[318] Pang L, Zhao X, Liu W, Deng J, Tan X, Qiu L (2015). Anticancer effect of ursodeoxycholic acid in human oral squamous carcinoma HSC-3 cells through the caspases. Nutrients.

[319] Fimognari C, Lenzi M, Cantelli-Forti G, Hrelia P (2009). Apoptosis and modulation of cell cycle control by bile acids in human leukemia T cells. Ann N Y Acad Sci.

[320] Wu YC, Chiu CF, Hsueh CT, Hsueh CT (2018). The role of bile acids in cellular invasiveness of gastric cancer. Cancer Cell Int.

[321] Lim SC, Duong HQ, Choi JE, Lee TB, Kang JH, Oh SH, Han SI (2011). Lipid raft-dependent death receptor 5 (DR5) expression and activation are critical for ursodeoxycholic acid-induced apoptosis in gastric cancer cells. Carcinogenesis.

[322] Lim SC, Duong HQ, Parajuli KR, Han SI (2012). Pro-apoptotic role of the MEK/ERK pathway in ursodeoxycholic acid-induced apoptosis in SNU601 gastric cancer cells. Oncol Rep.

[323] Lim SC, Han SI (2015). Ursodeoxycholic acid effectively kills drug-resistant gastric cancer cells through induction of autophagic death. Oncol Rep.

[324] Peng S, Huo X, Rezaei D, Zhang Q, Zhang X, Yu C, Asanuma K, Cheng E, Pham TH, Wang DH, Chen M, Souza RF, Spechler SJ (2014). In Barrett's esophagus patients and Barrett's cell lines, ursodeoxycholic acid increases antioxidant expression and prevents DNA damage by bile acids. Am J Physiol Gastrointest Liver Physiol.

[325] Abdel-Latif MM, Inoue H, Reynolds JV (2016). Opposing effects of bile acids deoxycholic acid and ursodeoxycholic acid on signal transduction pathways in oesophageal cancer cells. Eur J Cancer Prev.

[326] Goldman A, Condon A, Adler E, Minnella M, Bernstein C, Bernstein H, Dvorak K (2010). Protective effects of glycoursodeoxycholic acid in Barrett's esophagus cells. Dis Esophagus.

[327] Im E, Akare S, Powell A, Martinez JD (2005). Ursodeoxycholic acid can suppress deoxycholic acid-induced apoptosis by stimulating Akt/PKB-dependent survival signaling. Nutr Cancer.

[328] Saeki T, Yui S, Hirai T, Fujii T, Okada S, Kanamoto R (2012). Ursodeoxycholic acid protects colon cancer HCT116 cells from deoxycholic acid-induced apoptosis by inhibiting apoptosome formation. Nutr Cancer.

[329] Peiró-Jordán R, Krishna-Subramanian S, Hanski ML, Lüscher-Firzlaff J, Zeitz M, Hanski C (2012). The chemopreventive agent ursodeoxycholic acid inhibits proliferation of colon carcinoma cells by suppressing c-Myc expression. Eur J Cancer Prev.

[330] Shah SA, Volkov Y, Arfin Q, Abdel-Latif MM, Kelleher D (2006). Ursodeoxycholic acid inhibits interleukin beta 1 and deoxycholic acid-induced activation of NF-κB and AP-1 in human colon cancer cells. Int J Cancer.

[331] Feldman R, Martinez JD (2009). Growth suppression by ursodeoxycholic acid involves caveolin-1 enhanced degradation of EGFR. Biochem Biophys Acta.

[332] Kim YH, Kim JH, Kim BG, Lee KL, Kim JW, Koh S-J (2019). Tauroursodeoxycholic acid attenuates colitis-associated colon cancer by inhibiting nuclear factor kappaB signaling. J Gastroenterol Hepatol.

[333] Alpini G, Kanno N, Phinizy JL, Glaser S, Francis H, Taffetani S, LeSage G (2004). Tauroursodeoxycholate inhibits human cholangiocarcinoma growth via Ca 2+-, PKC-, and MAPK-dependent pathways. Am J Physiol Gastrointest Liver Physiol.

[334] Alasmael N, Mohan R, Meira LB, Swales KE, Plant NJ (2016). Activation of the Farnesoid X-receptor in breast cancer cell lines results in cytotoxicity but not increased migration potential. Cancer Lett.

[335] Sun J, Mustafi R, Cerda S, Chumsangsri A, Xia YR, Li YC, Bissonnette M (2008). Lithocholic acid down-regulation of NF-kappaB activity through vitamin D receptor in colonic cancer cells. J Steroid Biochem Mol Biol.

[336] Vogel SM, Bauer MR, Joerger AC, Wilcken R, Brandt T, Veprintsev DB, Rutherford TJ, Fersht AR, Boeckler FM (2012). Lithocholic acid is an endogenous inhibitor of MDM4 and MDM2. Proc Natl Acad Sci USA.

[337] Powell AA, LaRue JM, Batta AK, Martinez JD (2001). Bile acid hydrophobicity is correlated with induction of apoptosis and/or growth arrest in HCT116 cells. Biochem J.

[338] Qiao D, Im E, Qi W, Martinez JD (2002). Activator protein-1 and CCAAT/enhancer-binding protein mediated GADD153 expression is involved in deoxycholic acid-induced apoptosis. Biochem Biophys Acta.

[339] Lin R, Zhan M, Yang L, Wang H, Shen H, Huang S, Huang X, Xu S, Zhang Z, Li W, Liu Q, Shi Y, Chen W, Yu J, Wang J (2020). Deoxycholic acid modulates the progression of gallbladder cancer through N(6)-methyladenosine-dependent microRNA maturation. Oncogene.

[340] Pyo JS, Ko YS, Kang G, Kim DH, Kim WH, Lee BL, Sohn JH (2015). Bile acid induces MUC2 expression and inhibits tumor invasion in gastric carcinomas. J Cancer Res Clin Oncol.

[341] Krishnamurthy K, Wang G, Rokhfeld D, Bieberich E (2008). Deoxycholate promotes survival of breast cancer cells by reducing the level of pro-apoptotic ceramide. Breast Cancer Res.

[342] Yoon JH, Higuchi H, Werneburg NW, Kaufmann SH, Gores GJ (2002). Bile acids induce cyclooxygenase-2 expression via the epidermal growth factor receptor in a human cholangiocarcinoma cell line. Gastroenterology.

[343] Baek MK, Park JS, Park JH, Kim MH, Kim HD, Bae WK, Chung IJ, Shin BA, Jung YD (2010). Lithocholic acid upregulates uPAR and cell invasiveness via MAPK and AP-1 signaling in colon cancer cells. Cancer Lett.

[344] Debruyne PR, Bruyneel EA, Karaguni IM, Li X, Flatau G, Muller O, Zimber A, Gespach C, Mareel MM (2002). Bile acids stimulate invasion and haptotaxis in human colorectal cancer cells through activation of multiple oncogenic signaling pathways. Oncogene.

[345] Halvorsen B, Staff AC, Ligaarden S, Prydz K, Kolset SO (2000). Lithocholic acid and sulphated lithocholic acid differ in the ability to promote matrix metalloproteinase secretion in the human colon cancer cell line CaCo-2. Biochem J.

[346] Nguyen TT, Lian S, Ung TT, Xia Y, Han JY, Jung YD (2017). Lithocholic acid stimulates IL-8 expression in human colorectal cancer cells via activation of Erk1/2 MAPK and suppression of STAT3 activity. J Cell Biochem.

[347] Nguyen TT, Ung TT, Li S, Lian S, Xia Y, Park SY, Do Jung Y (2019). Metformin inhibits lithocholic acid-induced interleukin 8 upregulation in colorectal cancer cells by suppressing ROS production and NF-kB activity. Sci Rep.

[348] Cheng K, Raufman J-P (2005). Bile acid-induced proliferation of a human colon cancer cell line is mediated by transactivation of epidermal growth factor receptors. Biochem Pharmacol.

[349] Payne CM, Weber C, Crowley-Skillicorn C, Dvorak K, Bernstein H, Bernstein C, Holubec H, Dvorakova B, Garewal H (2007). Deoxycholate induces mitochondrial oxidative stress and activates NF-κB through multiple mechanisms in HCT-116 colon epithelial cells. Carcinogenesis.

[350] Centuori SM, Gomes CJ, Trujillo J, Borg J, Brownlee J, Putnam CW, Martinez JD (1861). Deoxycholic acid mediates non-canonical EGFR-MAPK activation through the induction of calcium signaling in colon cancer cells. Biochem Biophys Acta.

[351] Zhu Y, Zhu M, Lance P (2012). Stromal COX-2 signaling activated by deoxycholic acid mediates proliferation and invasiveness of colorectal epithelial cancer cells. Biochem Biophys Res Commun.

[352] Pai R, Tarnawski AS, Tran T (2004). Deoxycholic acid activates beta-catenin signaling pathway and increases colon cell cancer growth and invasiveness. Mol Biol Cell.

[353] Li Z, Tanaka M, Kataoka H, Nakamura R, Sanjar R, Shinmura K, Sugimura H (2003). EphA2 Up-regulation induced by deoxycholic acid in human colon carcinoma cells, an involvement of extracellular signal-regulated kinase and p53-independence. J Cancer Res Clin Oncol.

[354] Milovic V, Teller IC, Murphy GM, Caspary WF, Stein J (2001). Deoxycholic acid stimulates migration in colon cancer cells. Eur J Gastroenterol Hepatol.

[355] Milovic V, Teller IC, Faust D, Caspary WF, Stein J (2002). Effects of deoxycholate on human colon cancer cells: apoptosis or proliferation. Eur J Clin Invest.

[356] Qiao D, Stratagouleas ED, Martinez JD (2001). Activation and role of mitogen-activated protein kinases in deoxycholic acid-induced apoptosis. Carcinogenesis.

[357] Qiao D, Gaitonde SV, Qi W, Martinez JD (2001). Deoxycholic acid suppresses p53 by stimulating proteasome-mediated p53 protein degradation. Carcinogenesis.

[358] Lee HY, Crawley S, Hokari R, Kwon S, Kim YS (2010). Bile acid regulates MUC2 transcription in colon cancer cells via positive EGFR/PKC/Ras/ERK/CREB, PI3K/Akt/IkappaB/NF-kappaB and p38/MSK1/CREB pathways and negative JNK/c-Jun/AP-1 pathway. Int J Oncol.

[359] Lechner S, Müller-Ladner U, Schlottmann K, Jung B, McClelland M, Rüschoff J, Welsh J, Schölmerich J, Kullmann F (2002). Bile acids mimic oxidative stress induced upregulation of thioredoxin reductase in colon cancer cell lines. Carcinogenesis.

[360] Lee DK, Park SY, Baik SK, Kwon SO, Chung JM, Oh E-S, Kim HS (2004). Deoxycholic acid-induced signal transduction in HT-29 cells: role of NF-kappa B and interleukin-8. Korean J Gastroenterol.

[361] Fu T, Coulter S, Yoshihara E, Oh TG, Fang S, Cayabyab F, Zhu Q, Zhang T, Leblanc M, Liu S, He M, Waizenegger W, Gasser E, Schnabl B, Atkins AR, Yu RT, Knight R, Liddle C, Downes M, Evans RM (2019). FXR regulates intestinal cancer stem cell proliferation. Cell.

[362] Casaburi I, Avena P, Lanzino M, Sisci D, Giordano F, Maris P, Catalano S, Morelli C, Andò S (2012). Chenodeoxycholic acid through a TGR5-dependent CREB signaling activation enhances cyclin D1 expression and promotes human endometrial cancer cell proliferation. Cell Cycle.

[363] Gao L, Lv G, Li R, Liu WT, Zong C, Ye F, Li XY, Yang X, Jiang JH, Hou XJ, Jing YY, Han ZP, Wei LX (2019). Glycochenodeoxycholate promotes hepatocellular carcinoma invasion and migration by AMPK/mTOR dependent autophagy activation. Cancer Lett.

[364] Liao M, Zhao J, Wang T, Duan J, Zhang Y, Deng X (2011). Role of bile salt in regulating Mcl-1 phosphorylation and chemoresistance in hepatocellular carcinoma cells. Mol Cancer.

[365] Zhou M, Qi Z, Zhao J, Liao M, Wen S, Manyi Y (2017). Phosphorylation of Bcl-2 plays an important role in glycochenodeoxycholate-induced survival and chemoresistance in HCC. Oncol Rep.

[366] Xie G, Wang X, Huang F, Zhao A, Chen W, Yan J, Zhang Y, Lei S, Ge K, Zheng X, Liu J, Su M, Liu P, Jia W (2016). Dysregulated hepatic bile acids collaboratively promote liver carcinogenesis. Int J Cancer.

[367] Shellman Z, Aldhahrani A, Verdon B, Mather M, Paleri V, Wilson J, Pearson J, Ward C, Powell J (2016). Bile acids: a potential role in the pathogenesis of pharyngeal malignancy. Clin Otolaryngol.

[368] Liu X, Chen B, You W, Xue S, Qin H, Jiang H (2018). The membrane bile acid receptor TGR5 drives cell growth and migration via activation of the JAK2/STAT3 signaling pathway in non-small cell lung cancer. Cancer Lett.

[369] Sharma R, Quilty F, Gilmer JF, Long A, Byrne AM (2017). Unconjugated secondary bile acids activate the unfolded protein response and induce golgi fragmentation via a src-kinasedependant mechanism. Oncotarget.

[370] Yen CJ, Izzo JG, Lee DF, Guha S, Wei Y, Wu TT, Chen CT, Kuo HP, Hsu JM, Sun HL, Chou CK, Buttar NS, Wang KK, Huang P, Ajani J, Hung MC (2008). Bile acid exposure up-regulates tuberous sclerosis complex 1/mammalian target of rapamycin pathway in Barrett's-associated esophageal adenocarcinoma. Can Res.

[371] Soma T, Kaganoi J, Kawabe A, Kondo K, Tsunoda S, Imamura M, Shimada Y (2006). Chenodeoxycholic acid stimulates the progression of human esophageal cancer cells: a possible mechanism of angiogenesis in patients with esophageal cancer. Int J Cancer.

[372] Prichard DO, Byrne AM, Murphy JO, Reynolds JV, O'Sullivan J, Feighery R, Doyle B, Eldin OS, Finn SP, Maguire A, Duff D, Kelleher DP, Long A (2017). Deoxycholic acid promotes development of gastroesophageal reflux disease and Barrett's oesophagus by modulating integrin-αv trafficking. J Cell Mol Med.

[373] Morrow DJ, Avissar NE, Toia L, Redmond EM, Watson TJ, Jones C, Raymond DP, Litle V, Peters JH (2009). Pathogenesis of Barrett's esophagus: bile acids inhibit the Notch signaling pathway with induction of CDX2 gene expression in human esophageal cells. Surgery.

[374] Burnat G, Majka J, Konturek PC (2010). Bile acids are multifunctional modulators of the Barrett's carcinogenesis. J Physiol Pharmacol.

[375] Zhang R, Yin X, Shi H, Wu J, Shakya P, Liu D, Zhang J (2014). Adiponectin modulates DCA-induced inflammation via the ROS/NF-kappa B signaling pathway in esophageal adenocarcinoma cells. Dig Dis Sci.

[376] Roesly HB, Khan MR, Chen HDR, Hill KA, Narendran N, Watts GS, Chen X, Dvorak K (2012). The decreased expression of Beclin-1 correlates with progression to esophageal adenocarcinoma: The role of deoxycholic acid. Am J Physiol Gastrointest Liver Physiol.

[377] Huo X, Juergens S, Zhang X, Rezaei D, Yu C, Strauch ED, Wang JY, Cheng E, Meyer F, Wang DH, Zhang Q, Spechler SJ, Souza RF (2011). Deoxycholic acid causes DNA damage while inducing apoptotic resistance through NF-κ{green}b activation in benign barrett's epithelial cells. Am J Physiol Gastrointest Liver Physiol.

[378] Jenkins GJS, D'Souza FR, Suzen SH, Eltahir ZS, James SA, Parry JM, Griffiths PA, Baxter JN (2007). Deoxycholic acid at neutral and acid pH, is genotoxic to oesophageal cells through the induction of ROS: The potential role of anti-oxidants in Barrett's oesophagus. Carcinogenesis.

[379] Jenkins GJS, Cronin J, Alhamdani A, Rawat N, D'Souza F, Thomas T, Eltahir Z, Griffiths AP, Baxter JN (2008). The bile acid deoxycholic acid has a non-linear dose response for DNA damage and possibly NF-κB activation in oesophageal cells, with a mechanism of action involving ROS. Mutagenesis.

[380] Song S, Guha S, Liu K, Buttar NS, Bresalier RS (2007). COX-2 induction by unconjugated bile acids involves reactive oxygen species-mediated signalling pathways in Barrett's oesophagus and oesophageal adenocarcinoma. Gut.

[381] Hu Y, Jones C, Gellersen O, Williams VA, Watson TJ, Peters JH (1960). Pathogenesis of Barrett esophagus: deoxycholic acid up-regulates goblet-specific gene MUC2 in concert with CDX2 in human esophageal cells. Arch Surg.

[382] Wu JT, Gong J, Geng J, Song YX (2008). Deoxycholic acid induces the overexpression of intestinal mucin, MUC2, via NF-kB signaling pathway in human esophageal adenocarcinoma cells. BMC Cancer.

[383] Looby E, Abdel-Latif MMM, Athié-Morales V, Duggan S, Long A, Kelleher D (2009). Deoxycholate induces COX-2 expression via Erk1/2-, p38-MAPK and AP-1-dependent mechanisms in esophageal cancer cells. BMC Cancer.

[384] Chen M, Ye A, Wei J, Wang R, Poon K (2020). Deoxycholic acid upregulates the reprogramming factors KFL4 and OCT4 through the IL-6/STAT3 pathway in esophageal adenocarcinoma cells. Technol Cancer Res Treat.

[385] Xu Y, Feingold PL, Surman DR, Brown K, Xi S, Davis JL, Hernandez J, Schrump DS, Ripley RT (2017). Bile acid and cigarette smoke enhance the aggressive phenotype of esophageal adenocarcinoma cells by downregulation of the mitochondrial uncoupling protein-2. Oncotarget.

[386] Joshi S, Cruz E, Rachagani S, Guha S, Brand RE, Ponnusamy MP, Kumar S, Batra SK (2016). Bile acids-mediated overexpression of MUC4 via FAK-dependent c-Jun activation in pancreatic cancer. Mol Oncol.

